# Technology for monitoring everyday prosthesis use: a systematic review

**DOI:** 10.1186/s12984-020-00711-4

**Published:** 2020-07-14

**Authors:** Alix Chadwell, Laura Diment, M. Micó-Amigo, Dafne Z. Morgado Ramírez, Alex Dickinson, Malcolm Granat, Laurence Kenney, Sisary Kheng, Mohammad Sobuh, Robert Ssekitoleko, Peter Worsley

**Affiliations:** 1 University of Salford, Salford, UK; 2 People Powered Prosthetics Group, University of Southampton, Southampton, UK; 3 University College London, London, UK; 4 Exceed Research Network, Exceed Worldwide, Lisburn, UK; 5 Exceed Worldwide, Phnom Penh, Cambodia; 6 The University of Jordan, Amman, Jordan; 7 Makerere University, Kampala, Uganda

## Abstract

**Background:**

Understanding how prostheses are used in everyday life is central to the design, provision and evaluation of prosthetic devices and associated services. This paper reviews the scientific literature on methodologies and technologies that have been used to assess the daily use of both upper- and lower-limb prostheses. It discusses the types of studies that have been undertaken, the technologies used to monitor physical activity, the benefits of monitoring daily living and the barriers to long-term monitoring, with particular focus on low-resource settings.

**Methods:**

A systematic literature search was conducted in PubMed, Web of Science, Scopus, CINAHL and EMBASE of studies that monitored the activity of prosthesis users during daily-living.

**Results:**

Sixty lower-limb studies and 9 upper-limb studies were identified for inclusion in the review. The first studies in the lower-limb field date from the 1990s and the number has increased steadily since the early 2000s. In contrast, the studies in the upper-limb field have only begun to emerge over the past few years. The early lower-limb studies focused on the development or validation of actimeters, algorithms and/or scores for activity classification. However, most of the recent lower-limb studies used activity monitoring to compare prosthetic components. The lower-limb studies mainly used step-counts as their only measure of activity, focusing on the amount of activity, not the type and quality of movements. In comparison, the small number of upper-limb studies were fairly evenly spread between development of algorithms, comparison of everyday activity to clinical scores, and comparison of different prosthesis user populations. Most upper-limb papers reported the degree of symmetry in activity levels between the arm with the prosthesis and the intact arm.

**Conclusions:**

Activity monitoring technology used in conjunction with clinical scores and user feedback, offers significant insights into how prostheses are used and whether they meet the user’s requirements. However, the cost, limited battery-life and lack of availability in many countries mean that using sensors to understand the daily use of prostheses and the types of activity being performed has not yet become a feasible standard clinical practice. This review provides recommendations for the research and clinical communities to advance this area for the benefit of prosthesis users.

## Introduction

The World Health Organization (WHO) provides a global estimate of 35–40 million people who require prosthetics and orthotics services [1], and this demand is increasing due to a range of factors. The growing and aging global population, along with the rising incidence of vascular-related diseases has led to high amputation rates [1–4]. Urbanisation contributes to an increasing number of traffic accidents, and conflict and war, particularly in the Middle East and East Africa, have caused high rates of traumatic injury leading to amputations [5].

Appropriate prostheses and supporting services can result in improved mobility, function, aesthetics and comfort for the user [6]. Consequently, prosthesis provision can improve the ability of a person with limb absence to generate an income and participate in education and social activities, increasing their quality of life [7]. Prosthesis use may also reduce the severity of some comorbidities, and the medical and support costs associated with them [8].

The WHO recommends that prosthetic service provision should take an integrated approach, which should include fitting the prosthesis, training the wearer, rehabilitation, community support and repair services [9]. However, the WHO also identified that access to prosthetic and orthotic services is a particular challenge, including a lack of service provision, problems with service delivery associated with inadequate staffing or training, lack of funding, and crucially a lack of data and evidence [10]. Indeed, evidence on the best ways to design and distribute prostheses to make them widely accessible, affordable and meet the users’ needs is lacking [11].

Prosthetic service and design decisions are predicated on the assumption that the prosthetic devices are worn and used in users’ everyday lives [12–14]. When user centred design principles are used, prosthetic devices are better matched to the user’s needs, leading to better functional outcomes [15]. However, there is currently limited objective data on how much and when prostheses are worn, what tasks they are used for, and what individual adjustments to the design of prostheses would increase their usefulness for the wearer in their environment.

To capture data on everyday use of prostheses, several clinical questionnaires and self-reported surveys have been proposed [16, 17]. Such tools typically ask the respondent to estimate prosthesis use (e.g. hours of wear per day) and/or the set of everyday activities performed (e.g. walking/sitting/community participation) [16, 17]. Questionnaires that rely on self-reported measures are time-consuming for clinicians to administer and for users to complete. They provide only summary data, and lack accuracy because they are limited by the users’ comprehension and interpretation of the questions, their social bias and their recall [13]. Further, in countries where access to secondary or higher education is limited, users’ literacy and numeracy levels can be a barrier to the efficacy of questionnaires. In order to evaluate how well a prosthesis is meeting users’ needs, it is essential that users’ voices are included in the assessment and that qualitative research methods are used. However, digital technologies that can objectively monitor the use of prostheses offer a complementary approach to these other assessment methods for understanding the effectiveness of different prosthetic designs, componentry, and rehabilitation and lifestyle interventions [18–21]. There is a large body of literature on activity monitoring, but little has looked at measuring the activity of people who use prostheses [22–24]. Previous reviews of studies that monitor the physical activity of people with limb absence have focused on the lower-limb and on the application of activity monitoring techniques, using sensors to compare prosthetic componentry [18], and assessing the physical and mental health benefits of physical activity [25].

This paper is intended to have general relevance to people who use prosthetic devices, but was motivated in particular by collaborative research towards addressing the global challenges around prosthetics services in low-resource settings and lower- and middle-income countries (hereafter grouped as *low-resource settings*). The review discusses the challenges around the assessment of prosthetic device use in low-resource communities. Whilst advanced movement analysis methods such as motion capture offer detailed insights into gait quality, such facilities are rarely available in low-resource settings and service providers report a greater need to assess more general patterns of longer-term prosthesis use [26, 27]. People may receive training to use their prosthesis in a clinic, but their continuing use after returning home is completely unknown, as is the potential change in physical behaviour which their prosthetic device may enable.

Therefore, this paper reviews the scientific literature on methodologies and technologies that have been used to assess the community-based, daily use of both upper- and lower-limb prostheses. It discusses the types of studies that have been undertaken, the technologies used to monitor physical activity, the benefits of monitoring daily living and the barriers to long-term monitoring.

## Methodology

### Search strategy

This systematic literature review employed a search of publications up to November 2019. Five databases (PubMed, Web of Science, Scopus, CINAHL and EMBASE) were used to search for relevant articles using three groups of keywords to collect all studies that monitored the activity of prosthesis users during daily-living.

#### Community-based activity

“daily living” OR “free living” OR “daily life” OR “real world” OR activit* OR mobility OR “prosthetic use” OR “home use” OR “real life” OR “daily use”.

AND

#### Population of interest

“artificial limb” OR “artificial leg” OR “artificial arm” OR (prosthe* OR amput* AND (limb OR leg OR arm OR hand OR wrist OR elbow OR foot OR ankle OR knee OR transradial OR trans-radial OR transhumeral OR trans-humeral OR transtibial OR trans-tibial OR transfemoral OR trans-femoral)).

AND

#### Sensor for monitoring activity

actimetry OR sensor OR monitor* OR “inertial measurement unit” OR IMU OR acceleromet* OR gyroscope OR magnetometer OR “global positioning system” OR GPS OR “step count” OR pedometer OR “cadence” OR “steps/” OR “steps per”.

### Study selection

EMA and LD undertook the systematic search. After removing duplicates, the title and abstract of each publication were reviewed to determine its relevance. Any papers that did not report first-hand on digitally monitoring the activities of prosthesis users in a community setting (i.e. outside the lab or clinic) were excluded. Conference abstracts that did not link to a full conference paper were also excluded. Where the relevance was not clear from the title and abstract, papers were read in full to determine inclusion. For each included paper, the reference lists and forward citation reports from each database were consulted in order to identify additional relevant articles that were not found in the automatic search. Many of the reasons for monitoring prosthesis use are also relevant to the field of orthotics and orthosis use. However, inclusion of papers associated with orthotics was beyond the scope of this review.

### Analysis of studies

The papers were categorised by study type (words in *italics* are used as category abbreviations):

- Studies that developed or validated activity monitoring devices, *algorithms* and/or metrics for activity description, intensity and/or classification.
- Studies that compared metrics reflecting activity levels to *clinical scores*.
- Studies that compared *interventions* (including prosthetic components and lifestyle interventions).
- Studies that compared predefined *populations*.

Information was collected from each included paper on the aim and main findings, the population, sample size, types of sensors, locations of sensors, duration of the assessment, types of activity detected and amount of prosthesis use. The analysis divides the level of limb absence into four types; below-knee, including trans-tibial, Syme’s and partial-foot (BK), above knee, including trans-femoral, knee and hip disarticulation (AK), below elbow, including trans-radial, wrist disarticulation and partial-hand (BE), and above-elbow, including trans-humeral, elbow and shoulder disarticulation and forequarter (AE).

For 25% of the included studies, a double-blinded triangulation method was used to complete the analysis. The papers were divided up between 9 of the authors so that the classification of the papers into type of study and information collected from each paper could be compared between reviewers, reducing the risk of bias. All papers authored by members of the team were reviewed by a minimum of two reviewers to minimise potential bias. There were few disagreements during the double-blinded process, and these all occurred in determining the primary category for papers that fitted into multiple categories. Therefore, LD and AD reassessed lower-limb papers that fitted into multiple categories and AC and LK reassessed the categories of the upper-limb papers to ensure a consensus was reached for all studies.

## Results

**Fig. 1:**
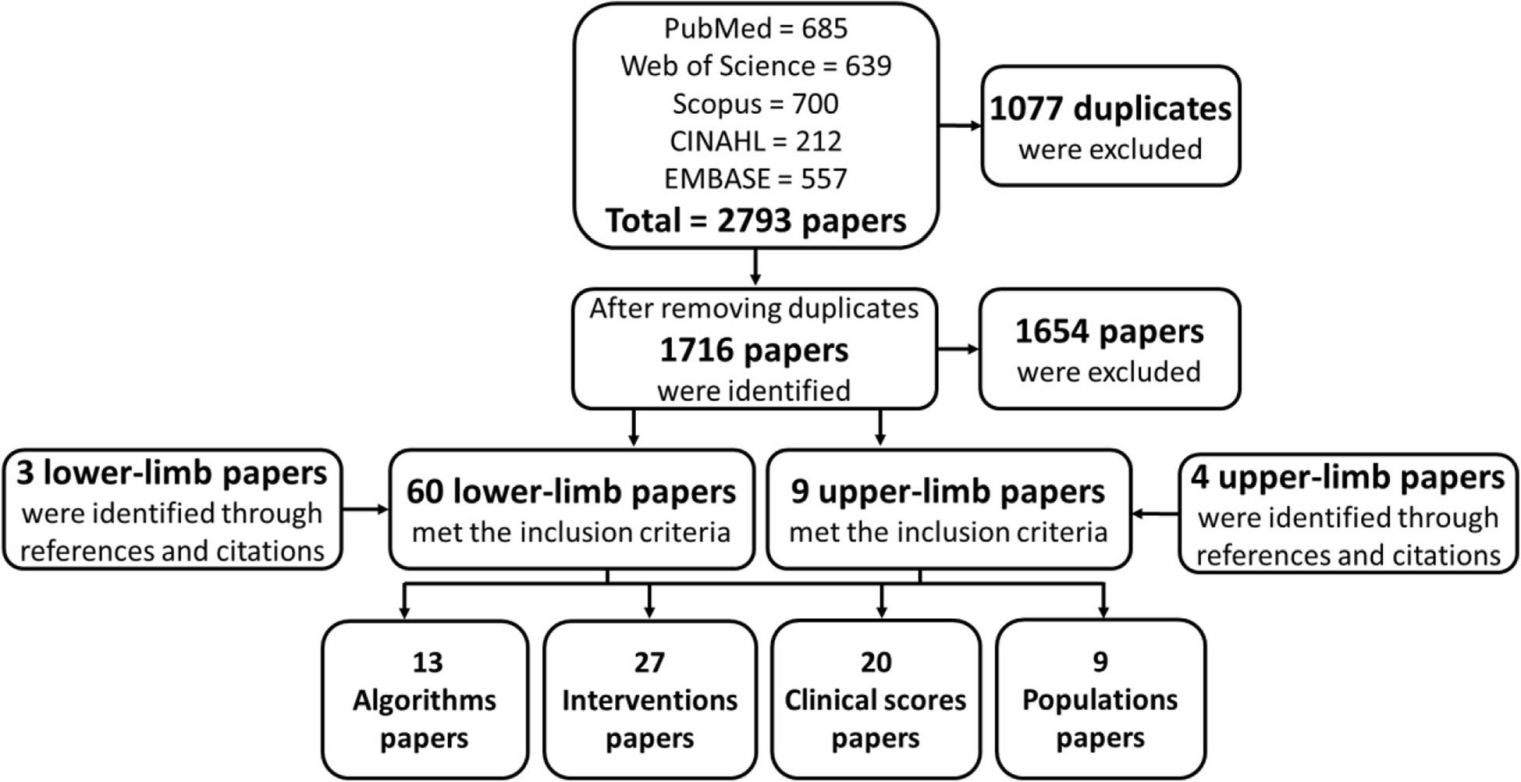
Flow chart of selection and sorting method

The search found 2793 papers across the 5 databases (Fig. 1). After removing duplicates, 1716 papers were screened by title and abstract. Of these, 57 lower-limb studies and 5 upper-limb studies were identified as relevant. An additional 3 lower-limb studies and 4 upper-limb studies were found through analysis of the references and citations of the included papers. Therefore, a total of 69 papers met the inclusion criteria, including one that was not in English, but was in French and able to be analysed by the authors [28].

Studies were categorised into the 4 topics, based on the primary reason for monitoring physical activity in the paper. Several articles addressed more than one of the topics for classification. In these cases, the authors conferred to find the main focus of the paper and placed the paper in that category. However, within this manuscript, where the body of evidence for each category is discussed, all papers that fitted the topic are included, even if it was not the primary focus of the paper. From the lower-limb studies, 43% focused on *interventions*, typically comparing prosthetic components. In contrast, the small number of upper-limb studies were fairly evenly spread between the following categories: *algorithms*, *clinical scores*, and *populations*.

Table 1 gives an overview of each paper included in the review, separated into topics. Many of the papers compared K-levels; a standard for classifying an individual’s functional status, as defined by Medicare [29].

**Table 1:** Overview of each paper included in the review. *Sensor locations: A = anatomical, P = prosthesis, L = liner.*

First author *(Date)*	Title	Sample *(Details)*	Sensors *(Locations)*	Activities identified *(Duration)*	Aim	Outcomes
**Developing or validating actimeters, algorithms and/or scores for activity classification**						
Arch (2017) [30]	Method to Quantify Cadence Variability of Individuals with Lower-Limb Amputation	*27 participants* *(10 BK and 1AK at K2, 10 BK and 6 AK at K3)*	Fitbit One *(P. Ankle)*	Activity level – steps, cadence *(7 days)*	Develop a method of quantifying real-world cadence variability.	This method of quantifying cadence variability can differentiate between K2 and K3 groups.
Arch (2018) [31]	Step count accuracy of StepWatch and FitBit One among individuals with a unilateral transtibial amputation	*50 participants* *(All BK)*	Fitbit One, StepWatch *(P. Ankle)*	Activity level – steps *(7 days)*	Compare step count accuracy of Fitbit One and StepWatch monitors during tasks and free-living.	Both monitors were accurate during forward-linear walking, but the StepWatch was more accurate during the Four Square Step Test and the Figure-of-8 Walk Test. The Fitbit counted fewer steps in free-living.
Belter *(2014)* [32]	Grasp force based taxonomy of split-hook prosthetic terminal devices	1 *participant* (BE)	Wide angle camera *(Forehead)*	Type of grasp and ability to exert force on the environment - prosthesis only *(5 h)*	Develop a taxonomy of split-hook grasping and force exertion during real life daily activities at home.	When using a split hook some types of grasp needed the help of the intact hand to pre-position the object. The ability to firmly grasp and hold objects was limited in both voluntary opening and closing hooks.
Chadwell (2018) [33]	Visualisation of upper limb activity using spirals - A new approach to the assessment of daily prosthesis usage	4 *participants* *(2 BE, 2 Anatomically Intact)*	2x ActiGraph GT3X+ *(A. and P. Wrists for prosthesis users, both A. Wrists for anatomically intact controls)*	Symmetry of non-specified arm movements - both arms Self-reported prosthesis wear time *(7 days)*	Propose a new method to analyse and visualise upper-limb activity data.	New approaches visualised the distribution of arm movements between the prosthetic and intact limb. A time series data spiral clearly illustrated arm activity over extended periods. The prosthesis users heavily relied on their intact limb.
Coleman (1999) [34]	Step activity monitor: Long-term, continuous recording of ambulatory function	3 participants *(2 BK, 1 Anatomically Intact with heart failure)*	Step Activity Monitor – SAM (later ‘StepWatch’) *(A. Ankle)*	Activity level – steps *(2 weeks)*	Provide guidelines and examples of using the Step Activity Monitor (SAM), and results of accuracy and reliability testing.	SAM is accurate, reliable, and can be used to perform long-term step monitoring on a range of subjects. It can quantify differences in ambulatory activity resulting from medical interventions and changes in health status.
Frossard (2008) [35]	Monitoring of the load regime applied on the osseointegrated fixation of a trans-femoral amputee: A tool for evidence-based practice	1 *participant* *(AK using osseo-integration)*	Transducer Model 45E15A *(P. Between residuum and knee)*	Loading and activity levels *(5 h)*	Describe the continuous recording of the true load regime experienced during daily living by the abutment of a person with a transfemoral amputation fitted with an osseo-integrated fixation.	The participant varied in lengths of time spent active, and averaged 64% of time spent inactive and 36% active, with an average 8 steps/min. Maximum load on the mediolateral, anteroposterior and long axes represented 21, 21 and 120% of the body weight, respectively.
Frossard (2010) [36]	Categorisation of activities of daily living of lower limb amputees during short term use of a portable kinetic recording system: a preliminary study.	1 *participant* *(AK using osseo-integration)*	Transducer Model 45E15A *(P. Between residuum and knee)*	Loading and activity levels *(5 h)*	Determine the relevance of the categorisation of the load data to assess the functional output and usage of the prosthesis.	Walking, localised movement, and standing occurred 44, 34 and 22% of recording time, respectively. The maximum forces on the mediolateral, anteroposterior and long axes were highest when walking and lowest when standing.
Hornero (2013) [37]	Bioimpedance system for monitoring muscle and cardiovascular activity in the stump of lower-limb amputees	5 *participants* *(All AK)*	Electrical impedance myography, electrical impedance plethys-mography *(A. Residuum)*	Breathing, muscle activity on the residual limb, heart-rate *(Not provided)*	Continuously monitor as many non-invasive physiological parameters as possible from people with lower-limb amputations using a single bioimpedance electrode configuration system.	The system monitors muscle activity, heart rate and breathing rate. The shape and amplitude of the changes in the electrical impedance myography signal correlated with the type of gait and the force exerted by the muscle.
Jayaraman (2014) [38]	Global Position Sensing and Step Activity as Outcome Measures of Community Mobility and Social Interaction for an Individual With a Transfemoral Amputation Due to Dysvascular Disease	1 *participant* *(AK)*	StepWatch 3.1, *(P.)* QStarz GPS *(A. pocket or purse)*	Steps, distance, speed, transport *(1 month)*	Objectively quantify community mobility and social interaction in an individual with AK amputation using a step activity monitor and GPS monitoring device over 1 month.	The method sensitively quantified community mobility and social activities, counting steps and recording the reasons the steps were taken, for insight into the participant’s everyday prosthesis use.
Sanders (2018) [39]	A Novel Method for Assessing Prosthesis Use and Accommodation Practices of People with Transtibial Amputation	21 *participants* *(All BK)*	WAFER –inductive sensor to find distance between liner and socket *(P. In socket)*	Donning and doffing *(8–13 days)*	Present a novel method for assessing prosthesis use and accommodation practices of people with transtibial amputation.	The WAFER showed good ability to detect donning and doffing but it had limited battery life and issues with alignment between the sensor on the socket and the target on the liner.
Shawen *(2017)* [40]	Fall Detection in Individuals With Lower Limb Amputations Using Mobile Phones: Machine Learning Enhances Robustness for Real-World Applications	17 participants *(7 AK, 10 Anatomically Intact)*	Samsung Galaxy S4 accelerometer and gyroscope *(A. Waist, pocket or in hand)*	Fall events *(2 days)*	Develop a fall-detection classifier that is robust to population, phone location and environmental sources of error, and detects falls for individuals with and without amputations.	The fall-detection classifier, trained using data from healthy anatomically intact individuals, was able to reliably separate falls from daily activities in individuals with AK amputation.
Spiers (2017) [41]	Analyzing at-home prosthesis use in unilateral upper-limb amputees to inform treatment & device design	3 participants *(2 BE, 1AE)*	GoPro Hero 3+ *(forehead)*	Type of grasp and grasp count - both arms *(Up to 4 h)*	Develop a taxonomy of manipulation suitable for use with unilateral upper-limb prosthesis users and demonstrate its use.	The taxonomy was applicable to the data from all 3 participants. Findings showed that intact hand use dominated across the 3 very different prosthesis users.
Stam *(1995)* [42]	A device for long-term ambulatory monitoring in trans-tibial amputees	1 participant *(BK)*	Continuous Ambulatory Monitoring of Prosthetic walking (CAMP) accelerometer *(P. Shank)*	Steps, walking periods *(5 days)*	Develop an actimeter that is lightweight, easily attaches to the prosthesis, and has energy and memory capacity for 5 days for practical clinical use.	The CAMP is lightweight and measures walking time of prosthesis users for up to 5 days. Further work will focus on decreasing size and weight, and increasing memory capacity.
**Comparing activity levels to clinical scores**						
Albert (2013) [43]	Monitoring Functional Capability of Individuals with Lower Limb Amputations Using Mobile Phones	18 participants *(10 AK, 8 Anatomically Intact)*	Android phone *(A. Lumbar spine)*	Activity level – thresholds of acceleration, *(7 days)*	Provide evidence that accelerometry, using mobile phones, can objectively quantify the activity levels of lower-limb prosthesis users.	K1 and K2 subjects are less active than the control subjects. K-levels co-vary with high level activity.
Albert (2014) [44]	Monitoring Daily Function in Persons With Transfemoral Amputations Using a Commercial Activity Monitor: A Feasibility Study	9 *participants* *(All AK)*	Fitbit One *(A. Wrist)*	Activity level – steps, distance *(7 days)*	Observe the relationship between the measured Fitbit activity levels and K-level classifications.	The percentage of movement time had a predictable relationship to the designated K-level. Activity level, measured outside the clinic, may lend support for K-level classifications.
Balkman (2019) [45]	Prosthetists’ perceptions of information obtained from a lower limb prosthesis monitoring system: a pilot study.	3 participants *(All AK)*	WAFER *(P. In socket, distal end)* 2x ActiGraph GT3X+ *(L. proximal to patella, P. lateral side of ankle)*	Donning and doffing, sitting, standing, walking *(2 weeks)*	Compare prosthetist-estimated patient activity with the prosthesis use and activity recorded by the sensors.	Prosthetists over- and under-estimated patient activity, relative to sensor data. Prosthetists found features of each presented survey tool to be clinically useful. Prosthesis-mounted monitors may provide prosthetists with improved understanding of their patients’ prosthesis use.
Chadwell (2018) [46]	Upper limb activity in myoelectric prosthesis users is biased towards the intact limb and appears unrelated to goal-directed task performance	40 participants *(20 BE, 20 Anatomically Intact)*	2x ActiGraphs from the GT3X range *(A. and P. Wrists for prosthesis users, both A. Wrists for anatomically intact controls)*	Symmetry of non-specified arm movements - both arms, self-reported and sensor calculate prosthesis wear time *(7 days)*	Report the activity of transradial myoelectric prosthesis users, and anatomically intact participants. Assess the extent to which self-report captures everyday patterns of activity, and whether kinematic and gaze-related measures of performance correlate with activity.	Prosthesis users relied heavily on their intact arm during everyday life, while intact adults demonstrated similar reliance on both arms. There was no correlation between the amount of time a prosthesis was worn and reliance on the intact limb, or between these measures and the kinematic and gaze-related measures of performance.
Cuberovic *(2019)* [47]	Learning of artificial sensation through long-term home use of a sensory-enabled prosthesis	1 participant *(BE with pre-implanted 8 channel flat interface nerve electrodes (FINEs))*	Aperture sensor, (P. base of thumb) 3x Flexiforce pressure sensors (P. thumb, index, and middle finger)	Grasp count – prosthesis only Hours of sensory feedback *(115 days)*	Study the effects of artificial sensory stimulation on perceptions related to the stimulation and functional outcomes, and assess correlation between use time and frequency of grasp use with clinical measures.	During the study, perception of sensation location and quality improved over time. As did prosthesis embodiment, confidence, and other psychosocial measures. Prosthesis usage did not increase, but was higher than in a previous study without sensory feedback.
Desveaux (2016) [48]	Physical Activity in Adults with Diabetes Following Prosthetic Rehabilitation	15 of 22 *participants* completed follow-up *(All BK)*	StepWatch *(A. Ankle)*	Activity level – steps *(5–9 days)*	Determine whether individuals with a BK amputation and diabetes meet guidelines for activity intensity and daily step counts, and whether clinical measures of physical function are associated with objective measures of physical activity.	Physical activity levels remained stable after discharge from rehabilitation but fell below recommended guidelines of steps per day and minutes of moderate to vigorous activity per week. Average activity levels correlated with clinical measures.
Godfrey *(2018)* [49]	The Accuracy and Validity of Modus Trex Activity Monitor in Determining Functional Level in Veterans with Transtibial Amputations	27 *participants* *(All BK)*	StepWatch and GPS *(A. Ankle)*	Activity level – steps *(10–16 days)*	Investigate the accuracy and reliability of Modus Trex–derived K-level to differentiate between Medicare Functional Classification levels (MCK-levels).	The Modus Trex–derived K-level was reliable and accurate at estimating MCK-levels and can be useful as a component in K-level evaluation.
Halsne (2013) [50]	Long-term activity in and among persons with transfemoral amputation	17 *participants* *(All AK: 6 at K2, 8 at K3, 3 at K4)*	StepWatch *(P. Ankle)*	Steps/day *(12 months)*	Objectively characterise the mobility dimension of participation in persons with AK amputation using long-term step activity data, and determine how activity varies over extended periods of time.	Activity between K2 and K3 subjects was not significantly different. Relative variation (CoV) was 0.65 across subjects but was lower for those with higher activity levels. Warmer seasons promoted higher activity, but peak temperatures and humidity reduced activity.
Kent *(2015)* [51]	Step activity and stride-to-stride fluctuations are negatively correlated in individuals with transtibial amputation	22 *participants* *(All BK at K3)*	ActiGraph *(P. Shank)*	Steps *(3 weeks)*	Determine whether increased stride-stride fluctuations also correspond to a reduced level of activity in daily life in people with BK amputation.	Stride-to-stride fluctuations correlated with decreased step counts, but it is unclear whether high fluctuations promoted decreased activity or less active individuals did not gain the experience to achieve skilled movement.
Lin (2014) [52]	Physical activity, functional capacity, and step variability during walking in people with lower-limb amputation	20 participants *(12 BK, 8 AK)*	Pedometer *(A. Waist)*	Steps *(7 days)*	Explore the associations between physical activity and physical performance measures (self-selected walking speed, 6-min walk test (6MWT), step length variability, and step width variability) in people with BK amputation.	Physical activity correlated with comfortable walking speed, 6MWT, and step width variability, and was inversely correlated with step length variability of the prosthetic leg and of the sound leg.
Mandel *(2016)* [53]	Balance confidence and activity of community-dwelling patients with transtibial amputation	22 participants *(All BK)*	StepWatch *(P. Ankle)*	Activity level – steps *(7 days)*	Examine the relationship between community-based physical activity and balance confidence in people with BK amputation who have low fall-risk.	There was a positive correlation between self-perceived balance confidence and community-based physical activity.
Orendurff (2016) [54]	Comparison of a computerized algorithm and prosthetists’ judgment in rating functional levels based on daily step activity in transtibial amputees	81 participants *(All BK)*	StepWatch *(P. Ankle)*	Activity level – steps *(7 days)*	Compare prosthetists’ ratings of K-levels based on a visual inspection of step activity patterns with the ratings calculated by an algorithm based on the same step activity data.	The algorithm produced functional level values that closely matched the average ratings of 14 experienced prosthetists. Linear regression indicated good linearity and concordance across the range of the two scales.
Orendurff *(2016)* [55]	Functional level assessment of individuals with transtibial limb loss: Evaluation in the clinical setting versus objective community ambulatory activity	12 participants *(All BK)*	StepWatch *(P. Ankle)*	Activity level – steps *(7 days)*	Determine the relationship between clinic-based K-level classification and K-level based on everyday ambulatory activity data collected by StepWatch.	There was good agreement between the two methods of determining K-level with R2 = 0.775 (*p* < 0.001).
Parker (2010) [56]	Ambulation of People With Lower-Limb Amputations: Relationship Between Capacity and Performance Measures	52 participants *(BK and AK, numbers not provided)*	StepWatch 3 *(P.)*	Activity level – steps *(7 days)*	Examine the relationship between clinical measures of ambulation capacity (using Locomotor Capabilities Index, 2MWT, and Timed Up and Go Test) and measures of ambulation performance in the community.	Capacity and performance showed moderate correlation (Spearman’s ρ: 0.41–0.78, *p* < 0.05). The highest correlation was the 2MWT and SAM peak activity index (0.78, *p* < 0.001). The 2MWT correlated with steps/day (*p* = 0.026) and TAPES (*p* = 0.016). Depressive symptoms correlated with decreased performance (*p* = 0.003, TAPES).
Pepin *(2019)* [57]	Correlation Between Functional Ability and Physical Activity in Individuals With Transtibial Amputations: A Cross-Sectional Study.	19 participants *(All BK. 5 at K2, 14 at K3/K4)*	ActivPAL *(A. Thigh)*	Activity – sitting/lying, standing, walking *(7 days)*	Examine the association between functional ability and physical activity in individuals with BK amputations.	Participants spent on average 19.7 h per day lying/sitting, 3.5 h standing, and 0.77 h walking. They walked an average of 3145 steps/day, placing them in the sedentary category.
Resnik *(2017)* [58]	The DEKA hand - A multifunction prosthetic terminal device - patterns of grip usage at home	21 participants *(Estimate ~ 40% AE ~ 60% BE, 1 bilateral – level unclear)*	On board software - not detailed - logs device on/off state, and motor and joint position data *(P.)*	Time prosthesis powered on, position of joint motors, time in each grip pattern. *(Up to 3 months)*	To quantify usage of DEKA hand grip patterns during home use and compare patterns of usage at home to test sessions.	The most used grips at home were power, pinch open, and lateral pinch. There were no differences in grip use over time. Power grip was used 55% of the time at home and 23% of the time during lab testing. Fewer grip patterns were used at home than in the lab.
Samuelsen (2017) [59]	The Impact of the Immediate Postoperative Prosthesis on Patient Mobility and Quality of Life after Transtibial Amputation	10 participants *(All BK)*	ActiGraph GT3X *(A. Belt buckle)*	Activity level – steps, energy expenditure *(10 days – 6 weeks)*	Measure activity using accelerometers, assess quality of life with the Medical Outcome Study Short Form-36, and Evaluate expected mobility status using the Amputee Mobility Predictor.	No significant relationships were observed between expected level of function and recorded activity level. Patients had low physical and emotional Short Form-36 component scores.
Sanders *(2018)* [60]	Residual limb fluid volume change and volume accommodation: Relationships to activity and self-report outcomes in people with trans-tibial amputation	29 participants *(All BK)*	ActiGraph GT3X+ *(P.)*	Activity - sitting, standing, walking, doffing *(3 h)*	Explore whether the morning-to-afternoon limb volume change was associated with time weight-bearing, satisfaction, comfort, or perceived mobility, and whether participants who changed sock thickness to adjust to volume change had better outcomes.	Factors other than time spent weight-bearing (standing and walking) correlated with the rate of morning-to-afternoon limb fluid volume change on BK prosthesis users.
Sions (2018) [61]	Self-Reported Functional Mobility, Balance Confidence, and Prosthetic Use Are Associated With Daily Step Counts Among Individuals With a Unilateral Transtibial Amputation	47 participants *(All BK)*	StepWatch *(P. Ankle)*	Activity level – steps *(7 days)*	Determine if self-reported measures, assessing constructs other than physical activity, are associated with accelerometer measurements of physical activity.	Clinical outcome measures may be predictive of daily physical activity as obtained with accelerometers among community ambulating longer-term BK prosthesis users.
Stepien (2007) [62]	Activity Levels Among Lower-Limb Amputees: Self-Report Versus Step Activity Monitor	77 participants *(All BK)*	StepWatch 3 *(P.)*	Activity level – steps *(8 days)*	Determine the accuracy of self-reported activity by community-dwelling, individuals with lower-limb amputation.	Participants averaged 3063 ± 1893 steps/day. Self-reported activity in an experimental setting was not accurate, and the measured and self-reported proportion of time spent in various states of activity showed poor agreement in rest, low, medium and high level activity. There was no bias toward either over- or under-reporting.
**Comparing interventions**						
Agrawal (2010) [63]	A comparison of gait kinetics between prosthetic feet during functional activities - Symmetry in External Work (SEW) approach	11 participants *(All BK)*	StepWatch *(Not provided)*	Activity level – steps (4* 10–14 days)	Validate a measure for quantifying gait differences among prosthetic feet. Calculate the reliability of the Symmetry in External Work (SEW) measure and determine its correlation with clinical measures.	The StepWatch results showed no difference in the number of steps or activity level of subjects. There was good correlation between the SEW values for level walking and other clinical outcome measures.
Andrysek (2017) [64]	Long-term clinical evaluation of the automatic stance-phase lock-controlled prosthetic knee joint in young adults with unilateral above-knee amputation	10 *participants* *(All AK)*	Power Walker EX-510 *(P. Anterior/ medial of thigh)*	Steps/day *(2 weeks)*	Compare the ASPL knee to the WBA knee on step count, walking speed and energy expenditure.	Participants did not tend to alter their walking speed or step count with the ASPL knee, but a reduction in energy expenditure was found.
Berge (2005) [65]	Efficacy of shock-absorbing versus rigid pylons for impact reduction in transtibial amputee	15 *participants* *(All BK)*	StepWatch *(P. Ankle)*	Activity level – steps (*7 days)*	Compare a shock-absorbing pylon to a rigid pylon to assess the effect on gait mechanics and functional outcomes using step counts and questionnaires.	No difference was found in number of steps/week. The only difference was that at initial contact, the prosthetic-side knee angle had more flexion with the rigid pylon while walking at a controlled speed (*p* = 0.004).
Buis (2014) [66]	Measuring the daily stepping activity of people with transtibial amputation using the ActivePAL activity monitor	48 *participants* *(All BK, 24 TSB sockets,* *24 PTB sockets)*	ActivPAL *(P. Ankle, anterior)*	Steps per minute/hour/day *(7 days)*	Compare the level of activity of prostheses users with PTB sockets to prostheses users with TSB sockets.	The differences in socket design did not result in significant differences in activity level.
Christiansen (2018) [67]	Behavior-Change Intervention Targeting Physical Function, Walking, and Disability After Dysvascular Amputation: A Randomized Controlled Pilot Trial	36 participants *(All BK)*	ActiGraph GT3X-BTa *(A. Waist)*	Activity level – steps *(10 days)*	Determine the efficacy of a home-based behaviour change intervention to promote exercise, and disease self-management after dysvascular transtibial amputation.	The home-based behaviour-change intervention improved daily step counts.
Coleman (2004) [68]	Quantification of prosthetic outcomes: Elastomeric gel liner with locking pin suspension versus polyethylene foam liner with neoprene sleeve suspension	13 *participants* *(All BK)*	StepWatch *(P. Ankle)*	Activity level – steps *(2 weeks)*	Compare two socket suspension systems: the Alpha liner with distal locking pin and the Pe-Lite liner with neoprene suspension sleeve, based on ambulatory activity, wear time, comfort and satisfaction.	10 participants preferred the Pe-Lite and 3 the Alpha. The Pe-Lite was worn for 82% more time and 83% more steps per day. Ambulatory intensity distribution did not differ and no differences were found from questionnaires.
Darter (2007) [69]	The effects of an integrated motor learning based treadmill mobility and aerobic exercise training program in persons with a transfemoral amputation	8 participants *(All AK)*	AMP 331 Accelerometer *(P. Ankle)*	Activity – steps, distance, speed (3* 7 days)	Determine daily averages for steps, speed and distance using an activity monitor before, during and after an 8-week mobility and aerobic exercise training intervention.	The mobility and aerobic exercise training was effective in improving gait performance, cardiorespiratory fitness, and locomotion related disability over the length of the intervention.
Gailey (2012) [70]	Application of self-report and performance-based outcome measures to determine functional differences between four categories of prosthetic feet	10 *participants* *(All BK - 5 with peripheral vascular disease (PVD), 5 without)*	Step Activity Monitor – SAM (later ‘StepWatch’) *(P. Ankle)*	Steps/day *(2 weeks)*	Determine the ability of self-report and performance-based measurements to detect functional differences between four categories of prosthetic feet, and whether differences exist between cohorts with and without peripheral vascular disease (PVD).	AMPPRO performance-based measure found differences between some feet from baseline (*p* < 0.05). No other differences were found between feet by performance-based measures (6MWT, SAM) or self-report measures (PEQ-13 and LCI). AMPRO and 6MWT found differences between the PVD and the non-PVD groups (*p* < 0.05) with the Proprio foot.
Graczyk (2018) [71]	Home use of a neural-connected sensory prosthesis provides the functional and psychosocial experience of having a hand again	2 participants *(Both BE with pre-implanted 8 channel flat interface nerve electrodes (FINEs))*	Aperture sensor, (P. base of thumb) 3x Flexiforce pressure sensors (P. thumb, index, and middle finger)	Grasp count – prosthesis only Hours of sensory feedback Self-reported prosthesis wear time *(36–47 days)*	Study the effect of electrical stimulation for sensory feedback while using a myoelectric prosthetic on use/non-use of the prosthesis, functional performance and psychosocial experience.	When sensory feedback was provided, participants used the prosthesis more, functional performance improved and psychosocial factors improved (self-efficacy, prosthetic embodiment, self-image, social interaction, and quality of life).
Hafner (2015) [72]	Physical performance and self-report outcomes associated with use of passive, adaptive, and active prosthetic knees in persons with unilateral, transfemoral amputation: Randomized crossover trial	12 *participants* (*All BK)*	StepWatch *(P. Ankle)*	Activity level – steps/day *(2–14 months)*	Assess and compare physical performance and self-reported outcomes that may be attributed to use of prosthetic knees with passive, adaptive, and active control in persons with unilateral BK amputation.	Compared with passive control, adaptive control improved comfortable Timed Up and Go (TUG) by 0.91 s (*p* = 0.001) and reported physical function by a T-score of 1.26 (*p* = 0.03), and active control increased comfortable TUG, fast TUG, and ramp times by 3.02, 2.66, and 0.96 s, respectively (all *p* < 0.03), and increased balance confidence by 3.77 (*p* = 0.003). Steps/day was lower when using the active knee than passive or adaptive knees.
Hafner (2007) [73]	Evaluation of function, performance, and preference as transfemoral amputees transition	17 *participants* *(All BK)*	StepWatch 2 *(P. Ankle)*	Steps/day, distance *(2 months)*	Evaluate differences in function, performance, and preference between mechanical and microprocessor prosthetic knee control technologies.	The microprocessor knee showed improved performance on stairs and hills, reduced frequency of stumbling, and was preferred by participants. No differences were found between knees on step frequency or estimated daily distance travelled.
Highsmith (2016) [74]	Effects of the Genium Knee System on functional level, stair ambulation, perceptive and economic outcomes in transfemoral amputees	20 *participants* *(All AK)*	StepWatch *(P. Ankle)*	Activity level – steps *(2 weeks)*	Determine if the Genium knee is beneficial, compared to the C-leg, using common clinical assessments.	The Genium knee improved stair walking, multi-directional stepping, functional level, steps/ day and perceived function, compared to the C-Leg.
Highsmith (2012) [75]	Spatiotemporal Parameters and Step Activity of a Specialized Stepping Pattern Used by a Transtibial Amputee During a Denali Mountaineering Expedition	1 *participant* *(BK)*	Sportline ThinQ XA Model 305 Pedometer *(A. Neck)*	Steps/day, cadence. *(8 days)*	Describe spatiotemporal differences between the French technique and typical walking patterns of the participant. Report the qualitative and quantitative step activity during a climbing expedition.	Stride, step, and double support times were greater in the French technique, but spatially, stride and step lengths were greater in the traditional stepping. Daily step count averaged 10,404 steps on active climbing days.
Hsu (2006) [76]	The Effects of Prosthetic Foot Design on Physiologic Measurements, Self-Selected Walking Velocity, and Physical Activity in People With Transtibial Amputation	8 *participants* *(All BK)*	Yamax Digiwalker Pedometer *(A. Iliac crest)*	Steps *(1 month)*	Investigate the physiological differences during treadmill walking and the physical activity profiles for the Otto Bock C-Walk foot (C-Walk), Flex-Foot, and solid ankle cushion heel (SACH) foot. Compare feet on step-count during daily physical activity.	The C-Walk trended towards better physiological responses compared with the SACH; however, no differences between feet were statistically significant. The Flex-Foot showed no differences in energy expenditure, gait efficiency, or steps/day, but showed a lower age-predicted maximum heart rate and perceived exertion.
Kaufman (2018) [12]	Functional assessment and satisfaction of transfemoral amputees with low mobility (FASTK2): A clinical trial of microprocessor-controlled vs. nonmicroprocessor- controlled knees	50 *participants* *(All AK)*	ActiGraph GT3X+ *(P and A. Waist, thigh, both ankles)*	Activity level – steps *(4 days)*	Assess if individuals with amputations categorised as K2 would benefit from a microprocessor-controlled knee.	Participants demonstrated a reduction in falls, less time spent sitting, and increased activity when using the microprocessor knee. They also reported better ambulation, improved appearance, and greater utility.
Klute (2006) [77]	Prosthetic Intervention Effects on Activity of Lower-Extremity Amputees	17 *participants* *(12 BK, 5 AK)*	StepWatch *(P. Ankle)*	Steps/day *(7 days)*	Investigate the effect of prosthetic interventions on the functional mobility of people with lower-limb amputation.	The intervention had no effect on activity level and duration. Individuals with BK amputation had higher activity levels on weekdays than weekends. Prosthetic components should be optimised for 1 to 2 min bouts of activity consisting of a few dozen steps.
Klute (2011) [78]	Vacuum-Assisted Socket Suspension Compared With Pin Suspension for Lower Extremity Amputees: Effect on Fit, Activity, and Limb Volume	5 *participants* *(All BK)*	StepWatch 3 *(P. Ankle)*	Activity level – steps *(2 weeks)*	Compare the fit and function of two socket and suspension systems: 1. A total surface-bearing socket with a vacuum-assisted suspension system (VASS), 2. A modified patellar tendon-bearing socket with a pin lock suspension system.	Activity levels were lower while wearing the vacuum-assisted socket suspension system than the pin suspension. The VASS socket fitted slightly better, as measured by pistoning.
Klute (2016) [79]	Prosthesis management of residual-limb perspiration with subatmospheric vacuum pressure	5 *participants* *(All BK)*	StepWatch *(P. Ankle)*	Activity level – steps *(2 weeks)*	Measure differences in activity levels, residual-limb skin temperatures, perspiration accumulation and expulsion, and subjective experiences between the dynamic air exchange (DAE) prosthesis and a standard-of-care total surface bearing suction socket.	During the 7-day acclimation, no difference in step activity levels was detected (*p* = 0.22). During the rest-walk-rest protocol, no differences in skin temperatures were observed (*p* = 0.37). The DAE prosthesis accumulated 1.09 ± 0.90 g and expelled 0.67 ± 0.38 g of perspiration. The suction prosthesis accumulated 0.97 ± 0.75 g. Participants were receptive to both prostheses.
Larson (2014) [80]	Massage therapy effects in a long-term prosthetic user with fibular hemimelia	1 *participant* *(BK)*	Pedometer Yamax SW-200 *(A. Hip on prosthetic side)*	Steps/day *(50 days)*	Evaluate the effectiveness of massage therapy to promote activity level, decrease low-back pain, and improve health-related quality of life (HRQOL) in a long-term prosthetic user with fibular hemimelia.	Pain level decreased, HRQOL increased, and no change occurred in ambulatory activity level.
Littman (2018) [81]	Pilot randomized trial of a telephone-delivered physical activity and weight management intervention for individuals with lower extremity amputation	15 *participants* *(12 BK, 3 AK)*	StepWatch *(Not provided)*	Steps *(7 days)*	Test the feasibility, acceptability, and safety of a weight management and physical activity intervention and obtain preliminary efficacy estimates for changes in weight, body composition, and physical functioning.	Coached participants had greater decreases in waist circumference and fat mass, but no significant intervention effects were observed for physical functioning or physical activity.
Morgan (2018) [82]	Laboratory- and community-based health outcomes in people with transtibial amputation using crossover and energy storing prosthetic feet: A randomized crossover trial	27 *participants* *(All BK)*	StepWatch *(P. Ankle)*	Activity level – steps *(1 month)*	Assess changes in lab-based endurance, perceived exertion and walking performance, and community-based step activity, self-reported mobility, fatigue, balance confidence, activity restrictions, and satisfaction for crossover and energy storing feet.	Self-reported results showed the users preferred crossover to energy storing devices. Quantitative measures did not show significant differences between prostheses, except for step length.
Raschke (2015) [83]	Biomechanical characteristics, patient preference and activity level with different prosthetic feet: A randomized double blind trial with laboratory and community testing	11 *participants* *(All BK)*	StepWatch *(P. Ankle)*	Activity level – steps *(7 days)*	Determine if reported preference was related to a biomechanical characteristic of different prosthetic feet, and if this resulted in increased community-based activity.	Each foot was evaluated over a similar number of steps, but no foot increased activity levels.
Sanders (2017) [84]	Effects of Socket Size on Metrics of Socket Fit in Trans-Tibial Prosthesis Users	9 *participants* *(All BK)*	ActiGraph GT3X *(P. Ankle)*	Steps *(2 weeks)*	Identify metrics of acceptable socket fit in people with BK amputation. Determine if a known change in prosthetic socket size was reflected in objective and subjective measures of fit, comfort, and performance.	Most promising variables for early detection of socket fit deterioration were step time and width asymmetry, anterior and anterior-distal morning-to-afternoon limb fluid volume change, SCS, and subscales of the PEQ.
Segal (2014) [85]	Does a Torsion Adapter Improve Functional Mobility, Pain, and Fatigue in Patients with Transtibial Amputation?	10 *participants* *(All BK)*	StepWatch 3 *(P. Shank – lateral side)*	Activity level – steps *(7 days)*	Determine if a torsion adapter results in improved functional mobility, pain and fatigue, compared to a rigid adapter.	For moderately active individuals with BK amputation the torsion adapter did not show improvements in functional mobility, pain or fatigue. However, small increases in low- and medium intensity activities with less pain were seen. The torsion adapter may benefit individuals who have difficulty navigating everyday environments.
Sherman (2018) [86]	Daily step count of British military males with bilateral lower limb amputations: A comparison of in-patient rehabilitation with the consecutive leave period between admissions	9 *participants* *(All bilateral, 4 AK/AK, 2 AK/BK, 1AK/BE, 1 AK/AE, 1 AK/BK/BE)*	Long-term Activity Monitor - LAM2 *(P. Thigh)*	Steps/day *(4 weeks)*	Characterise daily step counts of military personnel with bilateral lower-limb amputations due to trauma, and compare steps during and between in-patient rehabilitation intervals.	Mean daily step count decreased when away from rehabilitation.
Theeven (2012) [87]	Influence of advanced prosthetic knee joints on perceived performance of life activity	30 *participants* *(All AK at K2)*	ActiGraph GT1M *(A. Waist)*	Activity level – steps *(3 weeks)*	Investigate changes in perceived performance of K2 persons who transition from a mechanical- to a microprocessor-controlled prosthetic knee joint, and assess whether the transition between components affects the daily activity level.	Participants’ perception regarding ambulation and satisfaction with walking were higher with the microprocessor knee, but the activity level was similar on both knees.
Wurdeman (2017) [88]	Step Activity and 6-Minute Walk Test Outcomes When Wearing Low-Activity or High-Activity Prosthetic Feet	28 *participants* *(24 unilateral BK, 4 bilateral BK)*	Activity monitor *(P. Shank)*	Steps *(6 weeks)*	Determine changes in average daily step count and 6MWT due to use of low- and high-activity prosthetic feet, and examine the sensitivity of these measures to classify different feet.	Neither daily step count nor 6MWT were responsive to changes in prosthetic feet, so it is not recommended that these measures are used to assess outcomes for different prosthetic feet.
**Comparing populations**						
Arch (2016) [89]	Real-world walking performance of individuals with lower-limb amputation classified as Medicare Functional Classification Level 2 and 3	27 *participants* *(10 BK and 1 AK at K2, and 10 BK and 6 AK at K3)*	Fitbit One *(P. Ankle)*	Activity level – steps/day *(7 days)*	Investigate community walking performance measures (step count, amount of activity, and activity intensity) for individuals with unilateral lower-limb amputation classified as K2 and K3.	The K2 group had a slower self-selected walking speed, walked a shorter distance in 6 min, and had a lower total step count, than those classified as K3. The K2 group tended to spend more time in low-intensity activity and less time in high-intensity activity.
Bussmann (2004) [90]	Daily physical activity and heart rate response with a unilateral transtibial amputation for vascular disease	18 participants *(9 BK participants,* *9 anatomically intact controls)*	2 uniaxial, 1 biaxial ADX202 *(A. 2 on thigh,* *1 on chest)*	Lying/sitting/standing/walking/ cycling *(48 h)*	Assess whether individuals with unilateral transtibial amputation and vascular disease are less active than persons without known impairments.	Unilateral transtibial amputates with vascular disease were less active than persons without known impairments.
Carmona (2017) [28]	Walking activity in prosthesis-bearing lower-limb amputees	43 participants *(11 AK, 29 BK)*	StepWatch3 *(P. Ankle. If bilateral – on the side of the longer residuum)*	Activity level – steps, cadence *(15 days)*	Study walking activity in home-dwelling lower-limb prosthesis users, comparing amount of walking to age, weight, cause of amputation, time since amputation, level of amputation and use of a walking aid.	Individuals with BK amputation without vascular disease or a walking aid walked the most per day. Individuals with AK amputation walked 21% less, and those with walking aids walked 13% less. Body mass index did not correlate with time spent walking but did with walking speed.
Chadwell (2016) [91]	The reality of myoelectric prostheses - understanding what makes these devices difficult for some users to control	3 participants *(2 BE, 1 Anatomically Intact)*	2x ActiGraph GT3X+ *(A. and P. Wrists for prosthesis users, both A. Wrists for anatomically intact control)*	Symmetry and magnitude of non-specified arm movements - both arms Self-reported prosthesis wear time *(7 days)*	Demonstrate the feasibility of a protocol to understand the factors making myoelectric prostheses difficult to control. The activity monitoring part of the paper aimed to collect and analyse data from wrist worn monitors.	The anatomically intact participant was equally reliant on both arms, whilst the two prosthesis users were more reliant on their intact arm. The prosthesis users reported wearing their prostheses for 10h hours on days when it was worn, but one only wore the prosthesis on 3 out of 7 days.
Chadwell (2019) [92]	Upper limb activity of twenty myoelectric prosthesis users and twenty healthy anatomically intact adults	40 participants *(20 BE, 20 Anatomically Intact)*	2x ActiGraphs from the GT3X range. *(A. and P. Wrists for prosthesis users, both A. Wrists for anatomically intact controls)*	Raw accelerometer data, activity counts in 1 s and 60s epochs, self-reported wear and sleep diaries. Prosthesis non-wear algorithm. *(7 days)*	Report the dataset upon which Chadwell et al. [46] was based, making the data publicly available for secondary analysis by other researchers.	The raw accelerometry data, the activity count data, code for estimating wear/non wear and wear diaries from 20 myoelectric prosthesis users are provided.
Chu (2014) [93]	Comparison of prosthetic outcomes between adolescent transtibial and transfemoral amputees after Sichuan earthquake using Step Activity Monitor and Prosthesis Evaluation Questionnaire	21 *participants (11 BK, 10 AK)*	Stepwatch *(P. Ankle)*	Activity level – steps *(3 months)*	Compare the activity levels of adolescents with a transfemoral amputation to those with a transtibial amputation, and explore differences in prosthesis-related quality of life.	Adolescents with transfemoral amputation were less active than those with transtibial amputation. No differences were found on Prosthesis Evaluation Questionnaire outputs.
Hordacre (2015) [94]	Community activity and participation are reduced in transtibial amputee fallers: a wearable technology study	46 *participants* *(All BK)*	StepWatch 3 and GPS *(P. Ankle - lateral side)*	Steps *(7 days)*	Assess activity and participation characteristics in the home and various community settings for people with transtibial amputation who do and do not have a history of falling.	Participants with a history of falls demonstrated lower levels of community activity and participation. Activity levels were reduced for recreational and commercial roles.
Hordacre (2014) [95]	Use of an Activity Monitor and GPS Device to Assess Community Activity and Participation in Transtibial Amputees	46 *participants* *(All BK)*	StepWatch 3 and GPS *(P. Ankle - lateral side)*	Steps *(7 days)*	Assess wearable technology (step-counts and GPS) to measure community activity and participation, and whether participants with higher K-levels (as assessed by AMP-PRO and timed mobility measures) were more active in their community.	The study linked accelerometer and GPS data and found that individuals categorised as lower functioning (K1/2) showed lower community activity and participation than K3/4. No difference was found between K3 and K4 for community activity or participation.
Paxton (2016) [96]	Physical activity, ambulation, and comorbidities in people with diabetes and lower-limb amputation.	46 participants *(22 BK diabetics, 11 Anatomically Intact diabetics, 13 Anatomically Intact healthy)*	ActiGraph GT3X *(A. Waist)*	Activity level – steps *(10 days)*	Characterise physical activity and its relation to physical function and comorbidities in people with/without diabetes and amputation.	Physical activity was related to physical function in the diabetic intact-limb group and in the diabetic reduced-limb group, whereas no such relationship existed in the healthy group.

The 13 papers on developing or validating actimeters, *algorithms* or scores for activity classification for lower-limb prosthesis users mainly focused on development of sensors for monitoring activity [34, 35, 37, 39, 42]. However, they also included development of smart-phone software to monitor falls [40] or visualise gait [97] and papers on comparing sensors [31, 98], validating sensors [38] and validating classification methods [30]. In the case of upper-limb studies, three papers related to the development of *algorithms* for the assessment of activity [32, 33, 41]. Two of these papers reported on the use of head mounted cameras for the development of grasp taxonomies [32, 41]. The third paper reported on new techniques for the visualisation of data from wrist worn accelerometers, used to assess the relative levels of activity of the intact and prosthetic limbs [33].

The 27 papers on comparing *interventions* for lower-limb prosthesis users mostly compared prosthetic components, rather than comparing lifestyle interventions. Studies compared different sockets [66, 79, 84], suspension systems [68, 78], knees [12, 64, 72–74, 87], pylons [65] and feet [63, 70, 76, 82, 83, 88, 99]. One study compared knees and pylons [77], and one compared torsion and rigid adapters [85]. These studies focused mainly on the effect of prosthetic components with different mechanical characteristics from different manufacturers on the amount the prosthesis is worn, and the physical activity level of the wearer. None of the studies that compared prosthetic feet found significant differences between components. Likewise, most of the studies comparing other aspects of componentry (e.g. pylons and sockets) did not find significant differences with respect to the users’ performance between the different components analysed (measured using steps/day, various gait measures, falls, skin temperatures, lab-based performance measures or self-report measures). However, some of the studies on prosthetic knees showed differences between types, and the two studies that compared suspension systems found differences in wear-time, steps/day, pistoning, activity levels and user opinion. Only one *intervention* study was identified from the set of upper-limb studies and this used a log of grasping events to explore the effect of sensory feedback on use over time [71].

The lifestyle *interventions* for lower-limb prosthesis users were aimed at promoting physical activity [67, 69, 81], providing rehabilitation [86] or providing massage [80]. These all showed positive outcomes, from increased daily step counts [67] to weight loss [81] and decreased pain levels [100].

Seventeen papers compared activity levels of lower-limb prosthesis users to *clinical scores*. The most frequently used *clinical score* that was compared with measured everyday activity was the K-level score [30, 43, 44, 49–51, 54, 55, 57, 87, 89, 94, 95]. There was good correlation between the K-level score and the number of steps taken in everyday life [30, 43, 44, 49, 54, 55]. The general consensus was that monitoring participants in their daily life might provide a complementary analysis to better assess the capabilities and prosthesis requirements of individuals by providing activity levels, wear time and potentially gait characteristics, alongside subjective scores like satisfaction [30, 43, 44, 49, 50, 54, 55, 95]. The upper-limb papers that included a comparison to *clinical scores* were more varied in their focus. Two papers recorded the time that a sensory feedback system was powered on and assessed the effect on number of grasp events recorded, a variety of clinical scores and a selection of psychosocial and functional outcomes [47, 71]. One compared a participant’s selection of hand grips between a lab-based assessment and during everyday use [58]. In line with the findings of the lower-limb papers, the fourth upper-limb paper highlighted the complementary nature of the real-world assessment, emphasising how measures of everyday upper-limb symmetry and prosthesis wear time appear unrelated to performance measured using in-lab approaches [46].

The 7 papers comparing lower-limb prosthesis user *populations* ranged in topic. One compared those with an amputation to an anatomically intact control group [90], one compared those with diabetes to anatomically intact participants with and without diabetes [96], some studies compared different K-levels [89, 95], or amputation levels [28, 93], and one compared individuals with a history of falls to those without [94]. The main findings were that prosthesis users were less physically active than anatomically intact controls, and individuals with vascular disease, above-knee amputation or lower K-levels were less physically active, compared to those with traumatic injury, below-knee amputation, or higher K-levels, respectively. All 4 upper-limb papers which related to the *population* category were output as part of the same research study; these papers compared the symmetry of upper-limb activity between those with limb-absence and anatomically intact control participants [33, 46, 91, 92]. The results showed that the upper-limb activity of prosthesis users is heavily biased towards the anatomical limb, but in anatomically intact controls activity is quite evenly distributed between the dominant and non-dominant limbs.

The upper-limb studies varied in the types of sensor used for monitoring activity. These methods of assessing activity were grouped into four topics [1]: use of head-mounted video cameras to generate grasp taxonomies [32, 41] [2], use of wrist-worn accelerometers to measure aspects of symmetry in upper-limb activity and prosthesis wear time [33, 46, 91, 92] [3], use of on-board sensing to evaluate choice of grasp [58], and [4] use of on-board sensing to evaluate the use of a sensory feedback system and the number of grasp events [47, 71]. During the review process, five other studies evaluating the upper-limb activity of prosthesis users were identified, however these were excluded from the main review due to assessing all upper-limb activity (as opposed to just the use of the prosthesis) [101, 102], or because they were only undertaken as lab-based studies [103–105].

The majority of lower-limb studies used body-worn accelerometers. However, 5 used in-socket sensors [35–37, 39, 45], 4 used GPS in addition to accelerometers [38, 49, 94, 95], and 2 used phone-based accelerometers [40, 43]. The most commonly used actimeter in the lower-limb studies was the StepWatch, which counts steps but does not classify the type of activity. Some clinic-based studies included activity classification [13, 106, 107], but most community-based studies only assessed activity by counting steps per minute or per day. The studies that classified the type of activity, used activity monitors with sampling frequencies that ranged between 10 Hz and 60 Hz. The activities were typically classified using thresholding. The one study that compared classification techniques only addressed fall detection, rather than a range of physical activities [40].

**Fig. 2:**
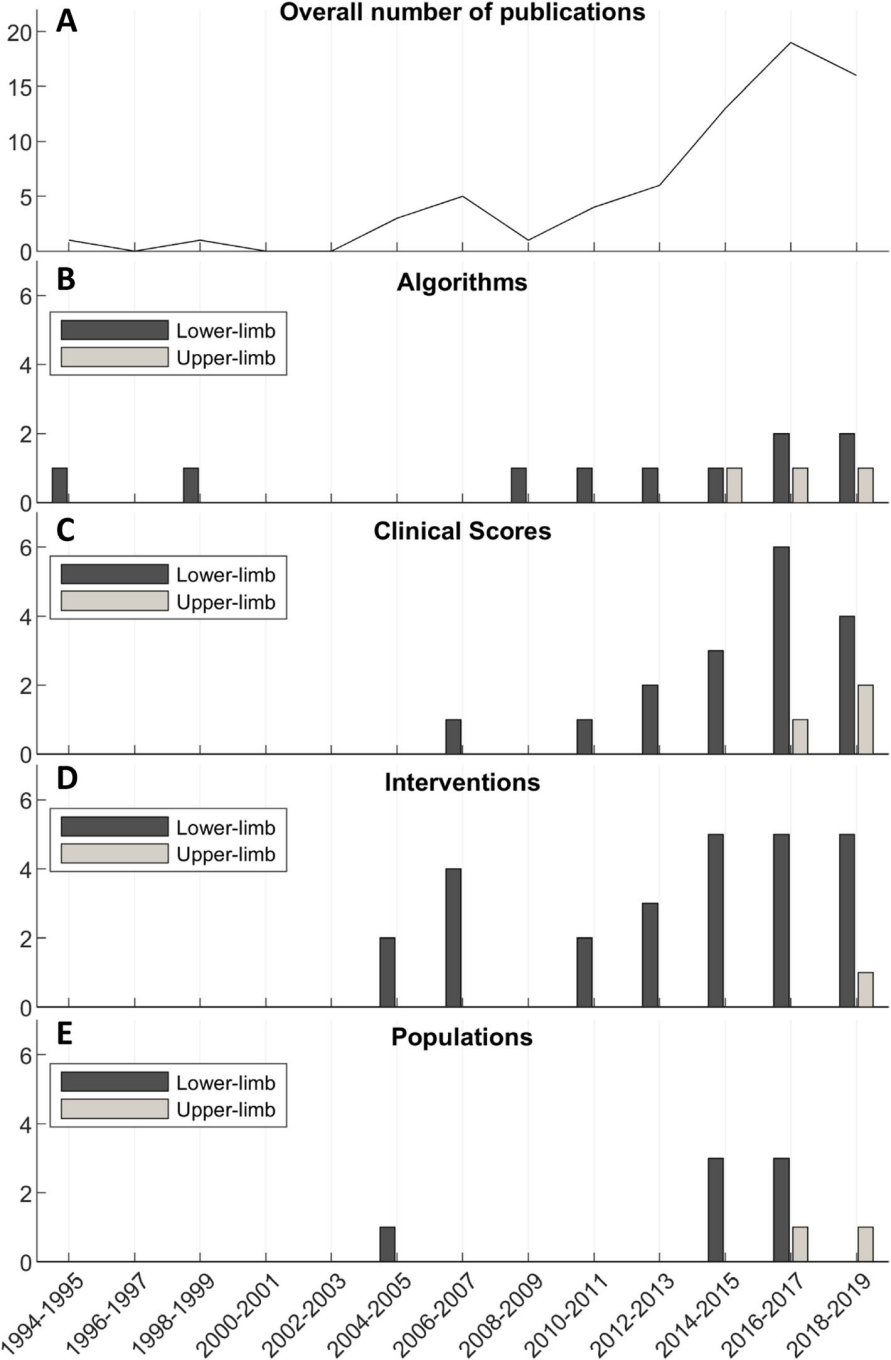
Number of publications per year (grouped into 2-year bins). **a** Overall number of publications, **(b**, **c**, **d** and **e**) Lower- and upper-limb publications separated into subplots by main topic

The number of publications relating to the digital monitoring of everyday prosthesis use has been gradually increasing over the past 25 years, with publications in the upper-limb field approximately 10 years behind those in the lower-limb field (Fig. 2). Unsurprisingly, papers discussing the development of *algorithms* were among the first to be published. However, it is interesting to note that papers relating to *interventions* came in very early in the lower-limb field, with *population*-based studies coming in more recently.

**Fig. 3:**
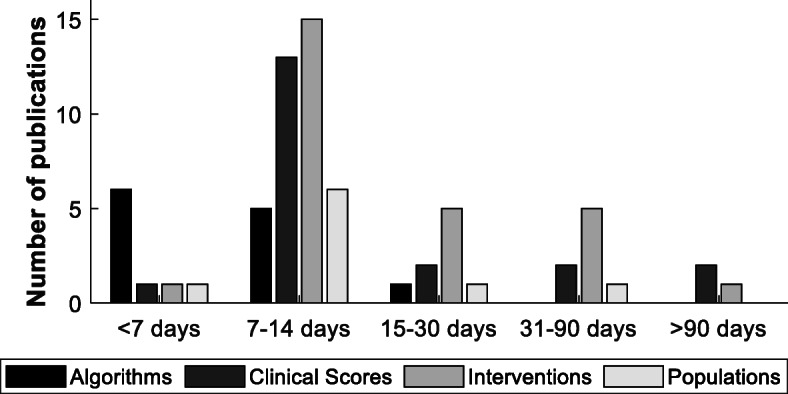
Recording period for studies split by main focus

Most studies recorded data for between one and 2 weeks (Fig. 3). Studies lasting for less than a week were generally those concentrating on the development of actimeters and *algorithms*, whilst studies lasting for more than 1 month were generally the *intervention*-based studies. Studies that compared activity monitoring to *clinical scores* or that compared *populations* typically used a 7-day protocol. Only three studies lasted for longer than 3 months.

## Discussion

This discussion focuses primarily on monitoring everyday lower-limb prosthesis use, representing the majority of the identified studies. However, with upper-limb studies starting to appear in the last decade, the focus of these are also discussed, particularly in relation to how the field may develop in the next decade in light of the trends in lower-limb research. It is worth noting that many of the findings from the lower-limb papers are also relevant to the upper-limb, for example, comments around sample size. The review’s motivation of community-based activity monitoring in low-resource settings is also addressed in each section, informed by the long-term experience of prosthetics service provision by our co-authors from Cambodia, Uganda and Jordan.

### Appraisal of studies by classification

#### Developing and validating actimeters, algorithms or scores for activity classification

There were few papers that developed ways to accurately monitor lower-limb prosthesis use with more detail than a simple step-count, and few that collected information on the types of activities being performed. This review shows that step detection methods have been well-established and are consistent across actimeters, though less accurate at low walking speeds [30, 108]. Step-count can be a useful indicator of exercise, but it does not provide information on the types of activities people with lower-limb absence can participate in and those that still have a barrier to access. Understanding the types of activity performed and whether someone is using transport (from accelerometer data) or leaving the house (from GPS data [38, 94, 95]) can provide an indication of community participation or isolation, which is often a significant issue for people with limb-absence, and can reveal information on physical functioning and gait quality [109, 110]. Body postures, such as sitting versus standing, also affect residual limb shape and volume. Understanding these changes better could improve socket fitting processes and the measurement of outcomes [111]. The ability to detect donning and doffing is also useful for understanding whether a prosthesis is meeting the user’s needs and may give more specific indications, such as physical and/or thermal comfort [39, 45, 112]. Changes in daily prosthesis wear time or the types of activity undertaken over time might provide an early warning of changes in socket fit and tissue health.

There are inertial measurement units that are pre-programmed to classify activities, such as the activPAL (PAL Technologies, Glasgow, UK) and the ActiGraph (ActiGraph LLC, Pensacola, USA). Studies have used these activity classification methods in a laboratory setting [13, 106, 107], but only a couple have used these methods to monitor activity in the community [45, 90]. These activity classification algorithms have mostly been designed for use with sensors worn on the thigh (ActivPAL), the wrist and the waist (studies have used the Actigraph at varying locations but the wrist and waist have the most validation). For long-term monitoring of prosthesis use, the authors suggest that embedding a sensor in the cosmesis of the prosthesis may enable longer monitoring periods, as it would remove the discomfort of having a sensor taped or strapped to the skin and the user would not need to remember to wear it, improving wear compliance. All prostheses can have a sensor attached to them, but not all have the capacity for the sensor to be embedded, given limited space available in the cosmesis. Certain types of sensors are bulky or have a wrist strap, which may make them unsuitable for embedding in the cosmesis or the wrong size to attach to locations other than the wrist. The lateral side of the shaft of a lower-limb prosthesis, just above the ankle, often has space to embed a sensor, and the authors have successfully embedded activPAL and Axivity sensors at this location on various styles of transtibial and transfemoral prosthesis. Attaching the sensor to a stable interface, such as the shank or socket rim minimises noise in the signal. It would be useful to develop algorithms that can detect the type of activity from a sensor located below the knee, so they can be used with below-knee prostheses. The ActiGraph has only a few studies that used the sensor on the ankle to classify activity, and these had low classification accuracy [113]. Sanders et al. [60] have presented some initial activity classification data from a sensor on the prosthetic ankle, whilst Redfield et al. [114] have also developed an algorithm for classifying activities from the prosthetic ankle, but it has not been tested in a daily-life setting [60, 90].

Considering upper-limb prostheses, prosthesis wear time is a key outcome, as if the user does not find the prosthesis to be of value, then it will not be worn. Detection of prosthesis wear/non-wear from accelerometry signals is complex and there is currently no validated upper-limb prosthesis non-wear algorithm. ActiGraph sensors offer two algorithms for the detection of sensor non-wear (“Troiano 2007” and “Choi 2011”), however both measures were developed based on data from hip-worn sensors and these would likely overestimate wear in the case of prostheses [115]. Chadwell et al. [46, 92] published a non-wear algorithm designed to detect prosthesis wear/non-wear using data collected from wrist-worn sensors and compared the calculated wear time to self-reported wear periods, however, this algorithm would require further validation before it is widely accepted. One of the main issues with detecting non-wear of a prosthesis is that the prosthesis can be removed and carried. Additional sensors such as a pressure, lux, or temperature transducer within the socket could offer a potential gold standard for prosthesis wear [105]. Until validated methods of automatically detecting aspects of prosthesis wear and use are available, self-report activity diaries offer an important complimentary measure.

It is important to note that the upper-limb papers reported data on the movements of the arms, or the number of different grasps performed over a specified period. Neither of these measures on their own provide a complete picture and an understanding of both when the prosthesis is worn and how much it is used. For an upper-limb prosthesis there are many aspects of use to consider, including: is the arm used? Are the arm movements similar to those of an anatomical arm or do they reflect compensatory movements? Are the active capabilities of the hand, such as grasping, being used and if so, to what extent? Although the field is in its infancy, many of these issues are beginning to be explored by different groups and hence there is great potential to combine techniques. For example, by combining accelerometry for the detection of arm movements with recordings of grip choice and frequency of use, comparisons could be made with studies of upper-limb activity in anatomically intact populations [116]. Comparing measures such as ‘system-on time’ against prosthesis wear time also make it possible to understand the value of advanced systems such as sensory feedback [47, 58]. It is worth noting that advanced multi-articulating upper-limb prostheses often log data on, for example, the grasps selected, or time powered up, and making these data available in a common and accessible format would help to move the field forward.

Accelerometers were the main type of sensor used in the studies to monitor physical activity. None of the studies monitored for more than a month without the participant returning to the clinic regularly to have the data downloaded, and there have been no longitudinal studies, such as studies that compare the level of activity of a first-time prosthesis user with their level of activity on their second or third prosthesis. Commercial accelerometer-based sensors are useful for research purposes, and the data recording capacity and battery life are increasing. However, they are still limited to a maximum of 3–6 months of recording time before the data needs to be downloaded and the battery recharged or replaced. These limitations, along with the expense of sensors, make them currently impractical for standard clinical use, particularly in low-resource settings. Cloud storage and inductive charging in areas with regular access to internet and reliable electricity may make long-term recording feasible.

#### Comparing prosthetic components and intervention strategies

A substantial portion (1/3) of the lower-limb studies focused on comparing prosthetic components. This is unsurprising, given the importance of increasing comfort and function of prostheses, along with improving access and affordability.

Most of these studies that compared prosthetic components did not find clinically significant results. This is not necessarily because there is no difference between the products, but is more likely because of the limited sample sizes, the wide variability between individuals that make it difficult to match controls, the limited time-frames for comparing results, and the insensitivity of the compared outputs (most only looked at step-count, not activity or gait quality). Small sample-size is a common issue in the field of prosthetics, as it is difficult to recruit large participant cohorts from the small limb-absent population [117]. One way to help address this issue is to develop a commonly-agreed framework for reporting participant characteristics, clinical outcomes and the engineering characteristics of the components tested, so that comparisons can be made across studies and the foundations laid for big data approaches [118]. Strengthening the partnerships and collaborations between academic institutions, the prosthetics industry, clinics, hospitals and societies of people with limb absence is important for ensuring research is informed by an understanding of the users’ needs, and that the research outputs inform changes in clinical practice. Having strong links within the prosthetics community, and empowering end-users to contribute to the research should also assist with recruitment.

Many of the studies ran the different interventions on the same participants so that each participant was their own control, to overcome the challenges of finding a well-matched control. However, this has its own limitations, as the order of testing the interventions can affect the outcomes, due to training effects, and the time of week or season might affect activity levels and types (i.e. due to weather, working patterns and religious practices such as Ramadan).

There were few studies that assessed lifestyle interventions for people with lower-limb absence, but the positive results, particularly in interventions to promote physical activity, demonstrate the importance of an inter-disciplinary approach to providing rehabilitation and community support, rather than simply providing the prosthetic device [67, 69, 80, 81, 86].

The role of inter-disciplinary rehabilitation begins before prosthetic fitting and continues after the prosthesis has been provided. Sensors that monitor non-use could also be useful for assessing issues with prostheses, to help identify where better training and/or support is needed, and to help prioritise where clinicians focus their efforts.

#### Comparing activity levels to clinical scores

In the lower-limb studies, the main clinical score that everyday activity levels were compared to was the K-level score [30, 43, 44, 49, 50, 54, 55, 95]. K-levels are the standard for classifying an individual’s current and potential functional status, particularly regarding ambulation. This classification was developed in 1995 and there is no gold standard method for establishing K-Levels [29, 119]. The common suite of tests used are clinic-based, providing information on the ability of the individual, rather than on their everyday functionality and needs. The ability level of the individual when ambulating in a clinical environment does not necessarily match how much they ambulate in their typical environment [55]. Nevertheless, the studies reviewed in this paper found that participants’ everyday physical activity mostly correlated with the K-level scores. However, monitoring participants in their daily life provided additional information that could complement the clinical measures to provide clinicians with a clearer picture of the individual’s capabilities and requirements. For example, the clinical K-level classifications were based on a physical examination at a single point in time, informed by clinical experience and subjective activity reports from the patient and family, whereas community activity monitoring increased the objectiveness in selecting suitable prosthetic components, adding a continuous element to assess changes in activity level over time [49, 54]. Community monitoring offers repeatable, objective criteria of functional level, based on the individual’s daily activities and environment.

#### Comparing different populations

The papers on comparing populations with lower-limb absence ranged in topic, but all demonstrated that there are significant differences between individuals and populations, so a one-size-fits-all method to providing prosthetics and services is not appropriate for meeting user needs. Of particular note in these studies was that people with vascular disease consistently showed lower levels of physical activity, so exercise-based interventions are possibly particularly important among this population. Few studies compared populations with BK limb absence to those with AK limb absence, but those that did found that individuals with AK limb absence walked fewer steps per day [93], walked for less time per day [28] and walked more slowly [52]. The characteristic of gait also differed, with AK individuals having a greater mean step width than those with BK limb absence [52]. Individuals with BK limb absence had higher activity levels on weekdays than weekends, but this difference was not observed in individuals with AK limb absence [77].

### Activity monitoring for low-resource settings

#### Benefits of activity monitoring in community-based settings

Ultimately, the measures of success for any prosthesis are whether the user chooses to wear it and use it to perform the functions it was designed for. In the case of lower-limb prostheses, the primary function is safe ambulation and in the upper-limb, it is prehensile function and the ability to locate and orient the prehensor in the reachable workspace. While there are many lab-based assessment studies investigating the extent to which particular prosthetic devices restore gait quality or upper-limb function, this review suggests that further work is needed to understand real-world needs and physical activity practices of prosthesis users and the factors which influence them.

In low-resource settings, public limb fitting centres and NGOs can only provide prostheses with basic functionality for a limited number of people annually, leaving hundreds or thousands of people waiting [1]. Furthermore, prostheses often require replacement after about 3–5 years due to wear and changes in residual limb size and shape. There are challenges worldwide around collecting meaningful measures of prosthetic rehabilitation outcomes, with some clinicians overwhelmed by their outcome measure workload and others performing only subjective evaluation of functional activity at the time of discharge [120]. Activity monitoring and gait assessment can perhaps provide direct measures of prosthesis use and therefore help the decision-maker to decide the appropriate type of prosthesis for an individual and the point at which to replace the prosthesis. Over a longer term, with larger datasets, service providers may use this approach to provide a cost effectiveness assessment of different prosthetic devices, and appraise new components.

Beyond amount of use, activity monitoring methods also offer an objective insight into how a prosthesis has been used. Currently, users retrospectively report about their experiences when they are seen by a healthcare professional. In low-resource settings, factors including a shortage of prosthetists and a lack of transport for those based in rural communities mean that it can be a long time before feedback is given to the provider [121]. Consequently, often users will remember only the very bad experiences, biasing their reports. Conversely, in some cultures service users will provide little negative feedback, especially where prosthetic devices have been given to them free of charge. In both situations, they may experience worsened musculoskeletal health and soft tissue injuries by needing to wear an ineffective prosthesis for longer. In some facilities, NGOs have developed community outreach programs where they provide assessment of prosthesis use among other services. If it was possible to monitor prosthesis use remotely, this could help to inform the decision-making process, and provide earlier intervention for users experiencing problems, as well as identifying devices with high rates of successful use. Real-time monitoring may even allow identification of users experiencing acute injury or mental health problems, evidenced by sudden changes in activity level or type [122, 123].

#### Barriers to activity monitoring

Despite the potential benefits of activity monitoring, barriers remain, associated with cost, access, training and capacity, as well as technical and cultural aspects of their use. Although commercial activity monitors are readily available in high resource settings, their cost-benefit balance must be considered in low-resource settings, especially with regard to widespread, real-time assessment. Adding embedded sensors to prostheses, and arranging for mobile or periodic connectivity may make them unaffordable for the services and users needing them most [124]. Furthermore, this would also require robust access to communication and information technology in low resource settings and for people with disabilities. Barriers to communication and challenges with accessing clinics (e.g. due to cost or availability of transport, and ability to take time off work) mean that in low-resource settings, losses to follow-up are likely to be more common. The cost associated with the potential loss of sensors may be particularly significant for clinics and researchers in low-resource settings. This emphasises the need for low cost appropriate monitoring technologies.

In both high and low-resource settings, many of the algorithms used to analyse gathered activity data are not user-friendly and require at least basic skills in programming and signal processing. Some fast, user-friendly analysis tools do exist, as in consumer activity monitors, but these represent an addition to busy clinicians’ workloads, where there are limited human resources and access to appropriate tools to undertake effective monitoring. More complex service-wide data analysis is in many instances time consuming, and requires specialist epidemiology training and statistics knowledge, which makes it impractical for clinical settings. Perceptions about how useful the information gathered from outcome measurements is for improving services for people with limb absence varies between countries, and countries that struggle to financially support systemic changes often see little value in gathering data on outcome measurements.

There is still a need to train clinicians in measuring outcomes, particularly in objective evidence assessment, multidisciplinary team integration and technology transfer. Client education is also essential for them to be able to participate fully and provide useful feedback, especially in low-resource settings. The training of clients must be accessible across varied literacy levels, as well as being culturally aware and co-designed using participatory research methods.

Evaluating prosthesis use in low-resource settings has challenges beyond access to measurement tools, limitations of current measuring tools, and the training of clinicians in how to use these tools. In Jordan, for instance, lower- and upper-limb prostheses are rarely evaluated, not due to the lack of awareness of the importance of the evaluation procedure, but rather due to the difficulty of implementing any rehabilitative intervention informed by the results of evaluation. The number of people with limb absence, and the lack of accessibility of limb fitting centres and of trained inter-disciplinary rehabilitation teams to deal with prosthetic training and problem solving were identified as key prosthetic and orthotic service access barriers by the WHO in 2011 [26, 125], and are issues in many countries.

Culture also affects the every-day use of activity monitors. It is important to have well-trained professionals and clinicians who understand the context and activities-of-daily-living of the assessed group to interpret the data. For example, considering activity types, the reviewed studies did not differentiate between social/community activities and work-related activities, nor did they evaluate the context of such activities. There are complex and nuanced links between disability, poverty and health [126]. In low-resource settings without social support systems, if a person is active because they must be active (work, school, child care, or other responsibilities), then activity may not relate to the function and comfort of the prosthesis. The reviewed studies showed diversity in location and type of data collected, but it would be useful to include in future studies the ethnography of participants to assess whether particular groups and lifestyles are more physically active, regardless of the prosthetic components available.

User-centred development of activity monitoring technology and methods must consider the prosthesis user as well as the clinician. In the present review, no articles reported on the user’s acceptability of the actimeters. It is important to understand the needs and ergonomic factors related to the use of actimeters. Monitoring tools which are bulky or not concealed within the prosthesis may be intrusive for users if they raise questions about what they are. Furthermore, monitoring an individual’s activities may be seen in some cultures as an invasion of privacy, so it is important for individuals to consent to what data is collected, how their data is used and to who has access to it [127, 128].

### Recommendations for future research utilising activity monitors to track prosthesis use

There have been few studies exploring psychological aspects, such as prosthesis embodiment [129], sensory preference [130] and attitudes of communities to disabilities [131] on wear and use of prostheses. It would, therefore, be useful to collect long-term data on community-based activities, particularly in regard to community participation and isolation, which is a common issue found amongst prosthesis users, and has been linked to quality of life scores [17, 132]. Physical activity monitoring in the community may also enhance knowledge of the links between physical activity and other factors, including prosthetic socket fit for comfort, function and reducing energy consumption. Socket fit plays a significant part in successful rehabilitation and restoration of function and mobility, but tools to objectively evaluate socket fit are lacking [133].

Most studies did not report on factors such as the weather, time-of-week, season or whether a walking aid was used. Factoring in these other aspects can provide greater understanding of the variations in an individual’s activity, and provide better support for clinical scores and prosthetists’ decisions. Some upper-limb studies assessed wear-time [33, 46, 91, 92], but most lower-limb studies did not. Wear-time, in addition to the total amount of activity, could give a better indication of whether there are issues with prosthesis comfort and whether users find a prosthesis beneficial in all situations, or only in certain situations (for example, many may use a prosthesis in public but not in private, or only outdoors, not indoors). The studies also did not report on durability or waterproofing of the sensors, which has particular relevance for sanitation chores, such as hand washing clothes [134], and for long-term-use assessment in rainy or humid environments.

Development of algorithms that allow sensors to provide detailed movement analytics, including information on gait symmetry, stability for safe ambulation, stride length, compensatory movements and upper-limb movement analytics could provide additional information to inform clinicians as they plan rehabilitation and exercises for prosthesis users, to increase prosthesis functionality. When selecting sensors to monitor physical activity, it is recommended by the authors that sensors are used that allow access to the raw data, as this enables bespoke data processing and study reproduction without the limitations of specific manufacturers.

Long-term monitoring of prosthesis-use and developing shared datasets supported by metadata standards may provide early warning of changes in socket fit and tissue health, enable comparisons to be made across studies to assist service providers in assessing prosthetic components, and help identify the unmet needs of prosthesis-users [135].

## Conclusion

This review has characterised scientific literature on methodologies and technologies that have been used to assess the community-based, daily use of upper- and lower-limb prostheses. The number of publications has increased over the past 25 years with publications on lower-limb being the primary focus, and upper-limb papers approximately 10 years behind. Research has utilised technology to assess step-counts as the primary measure of lower-limb prosthesis-user activity, and symmetry between the arm with the prosthesis and the intact arm for upper-limb prosthesis users. Community-based activity monitoring has been useful in evaluating prosthetic components and intervention strategies, comparing different populations and providing clinicians with a clearer picture of the individual’s capabilities and requirements than clinical measures alone.

The authors recommend a synchronised approach to developing a framework to monitor prostheses use outside the clinic that takes into account the daily life contexts in low-resource settings, where data on community-based prosthesis use is predominantly lacking. The field of prosthetics research would benefit from embracing technology for monitoring prosthesis use outside the clinic, as it can enable the formation of a framework and the accumulation of data and evidence that is necessary to design devices better matched to users’ needs and accounting for their real-life environments. Community-based activity monitoring of prosthesis users could provide many benefits for researchers, clinicians and end-users but the technology and current rehabilitation service systems still have barriers to long-term monitoring.

## Data Availability

The dataset supporting the conclusions of this article is available in the University of Southampton repository, 10.5258/SOTON/D1462.

## Abbreviations

2MWT: 2-min walk test
6MWT: 6-min walk test
AE: Above elbow (including elbow and shoulder disarticulation and forequarter)
AK: Above knee (including knee and hip disarticulation)
AMPRO: Amputee mobility predictor
BE: Below elbow (including wrist disarticulation and partial-hand)
BK: Below knee (including Syme’s and partial-foot)
DAE: Dynamic air exchange
GPS: Global positioning system
HRQOL: Health-related quality of life
LCI: Locomotor Capabilities Index
NGO: Non-governmental organisation
PEQ-13: Prosthesis Evaluation Questionnaire
PTB: Patellar tendon bearing
PVD: Peripheral vascular disease
SACH: Solid ankle cushion heel
SCS: Socket comfort score
SEW: Symmetry in External Work
TAPES: Trinity Amputation and Prosthesis Evaluation Scale
TSB: Total surface bearing
TUG: Timed up-and-go
VASS: Vacuum-assisted suspension system
WHO: World Health Organization

## References

[1] WHO (2017). Standards for prosthetics and orthotics.

[2] UN Department of Economic and Social Affairs. *World Population Prospects 2019, Online Edition. Rev. 1.* Geneva: United Nations; 2019.

[3] Dillon MP, Fatone S, Quigley M (2018). Uncertainty with long-term predictions of lower-limb amputation prevalence and what this means for prosthetic and orthotic research. J Prosthetics Orthot.

[4] WHO. *Global disability action plan 2014-2021: better health for all people with disability:* Geneva: World Health Organization; 2015.

[5] Fares J, Fares M, Fares Y (2020). Medical schools in times of war: integrating conflict medicine in medical education. Surg Neurol Int.

[6] Gallagher P, Desmond D (2007). Measuring quality of life in prosthetic practice: benefits and challenges. Prosthetics Orthot Int.

[7] Gallagher P, Donovan MO, Doyle A, Desmond D (2011). Environmental barriers, activity limitations and participation restrictions experienced by people with major limb amputation. Prosthetics Orthot Int.

[8] Sinha R, Van Den Heuvel WJA, Arokiasamy P (2011). Factors affecting quality of life in lower limb amputees. Prosthetics Orthot Int.

[9] MacLachlan M (2018). Systems thinking for assistive technology: redesigning the future. *ISPO UK annual scientific meeting*.

[10] Lemaire ED, Supan TJ, Ortiz M (2018). Global standards for prosthetics and orthotics. Can Prosthetics Orthot J.

[11] Borg J, Lindstrom A, Larsson S (2011). Assistive technology in developing countries: a review from the perspective of the convention on the rights of persons with disabilities. Prosthetics Orthot Int.

[12] Kaufman KR, Bernhardt KA, Symms K (2018). Functional assessment and satisfaction of transfemoral amputees with low mobility (FASTK2): a clinical trial of microprocessor-controlled vs. non-microprocessor-controlled knees. Clin Biomech (Bristol, Avon).

[13] Gardner DW, Redd CB, Cagle JC, Hafner BJ, Sanders JE (2016). Monitoring prosthesis user activity and doffing using an activity monitor and proximity sensors. J Prosthetics Orthot.

[14] Chamlian TR (2014). Use of prostheses in lower limb amputee patients due to peripheral arterial disease. Einstein (São Paulo).

[15] Sharp H, Preece J, Rogers Y. *Interaction design: beyond human-computer interaction.* 5th ed: Wiley; 2019.

[16] Heinemann AW, Ehrlich-Jones L, Connelly L, Semik P, Fatone S (2017). Enhancing quality of prosthetic services with process and outcome information. Prosthetics Orthot Int.

[17] Hawkins AT (2016). The effect of social integration on outcomes after major lower extremity amputation. J Vasc Surg.

[18] Balk EM (2018). Lower limb prostheses: measurement instruments, comparison of component effects by subgroups, and long-term outcomes. *Comparative effectiveness review: number 213. U.S. Department of Health and Human Services*.

[19] Williams RJ, Holloway C, Miodownik M (2016). The ultimate wearable: connecting prosthetic limbs to the IoPH. *UbiComp* 2016 *Adjunct - Proceedings of the 2016 ACM International Joint Conference on Pervasive and Ubiquitous Computing*.

[20] Hafner BJ, Sanders JE (2014). Considerations for development of sensing and monitoring tools to facilitate treatment and care of persons with lower-limb loss: a review. J Rehabil Res Dev.

[21] Prince SA (2008). A comparison of direct versus self-report measures for assessing physical activity in adults: a systematic review. Int J Behav Nutr Phys Act.

[22] Yang CC, Hsu YL (2010). A review of accelerometry-based wearable motion detectors for physical activity monitoring. Sensors..

[23] Uddin M, Salem A, Nam I, Nadeem T (2015). Wearable sensing framework for human activity monitoring. In WearSys 2015 - proceedings of the 2015 workshop on wearable systems and applications.

[24] Mukhopadhyay SC (2015). Wearable sensors for human activity monitoring: a review. IEEE Sensors J.

[25] Pepin ME, Akers KG, Galen SS (2018). Physical activity in individuals with lower extremity amputations: a narrative review. Phys Ther Rev.

[26] WHO. *World report on disability. *Geneva: World Health Organisation; 2011.

[27] Dickinson A, et al. Technologies to enhance quality and access to Prosthetics & Orthotics: the importance of a multidisciplinary, user-centred approach. *Global Report on Assistive Technology (GReAT) Consultation;* Geneva: World Health Organisation; 2019.

[28] Carmona G-A, Lacraz A, Assal M (2007). Walking activity in prosthesis-bearing lower-limb amputees. Rev Chir Orthop Reparatrice Appar Mot.

[29] Gailey RS (2002). The amputee mobility predictor: an instrument to assess determinants of the lower-limb amputee’s ability to ambulate. Arch Phys Med Rehabil.

[30] Arch ES (2017). Method to quantify cadence variability of individuals with lower-limb amputation. J Prosthetics Orthot.

[31] Arch ES, Sions JM, Horne J, Bodt BA (2018). Step count accuracy of StepWatch and FitBit one among individuals with a unilateral transtibial amputation. Prosthetics Orthot Int.

[32] Belter JT, Reynolds BC, Dollar AM. Grasp and force based taxonomy of split-hook prosthetic terminal devices. *36th Annual International Conference of the IEEE Engineering in Medicine and Biology Society*; 2014:6613–8. 10.1109/EMBC.2014.6945144 25571512

[33] Chadwell A (2018). Visualisation of upper limb activity using spirals: a new approach to the assessment of daily prosthesis usage. Prosthetics Orthot Int.

[34] Coleman KL, Smith DG, Boone DA, Joseph AW, Del Aguila MA (1999). Step activity monitor: long-term, continuous recording of ambulatory function. J Rehabil Res Dev.

[35] Frossard L (2008). Monitoring of the load regime applied on the osseointegrated fixation of a trans-femoral amputee: a tool for evidence-based practice. Prosthetics Orthot Int.

[36] Frossard L, Stevenson N, Sullivan J, Uden M, Pearcy M (2011). Categorisation of activities of daily living of lower limb amputees during short term use of a portable kinetic recording system: a preliminary study. J Prosthetics Orthot.

[37] Hornero G, Diaz D, Casas O (2013). Bioimpedance system for monitoring muscle and cardiovascular activity in the stump of lower-limb amputees. Physiol Meas.

[38] Jayaraman A, Deeny S, Eisenberg Y, Mathur G, Kuiken T (2014). Global position sensing and step activity as outcome measures of community mobility and social interaction for an individual with a transfemoral amputation due to dysvascular disease. Phys Ther.

[39] Sanders JE (2018). A novel method for assessing prosthesis use and accommodation practices of people with transtibial amputation. J Prosthetics Orthot.

[40] Shawen N (2017). Fall detection in individuals with lower limb amputations using Mobile phones: machine learning enhances robustness for real-world applications. JMIR mHealth uHealth.

[41] Spiers AJ, Resnik L, Dollar AM (2017). Analyzing at-home prosthesis use in unilateral upper-limb amputees to inform treatment & device design. IEEE Int Conf Rehabil Robot.

[42] Stam HJ, Eijskoot F, Bussmann JBJ (1995). A device for long term ambulatory monitoring in trans-tibial amputees. Prosthetics Orthot Int.

[43] Albert MV (2013). Monitoring functional capability of individuals with lower limb amputations using mobile phones. PLoS One.

[44] Albert MV, Deeny S, McCarthy C, Valentin J, Jayaraman A (2014). Monitoring daily function in persons with transfemoral amputations using a commercial activity monitor: a feasibility study. PM R.

[45] Balkman GS, Vamos AC, Sanders JE, Larsen BG, Hafner BJ (2019). Prosthetists’ perceptions of information obtained from a lower limb prosthesis monitoring system: a pilot study. J Prosthet Orthot.

[46] Chadwell A (2018). Upper limb activity in myoelectric prosthesis users is biased towards the intact limb and appears unrelated to goal-directed task performance. Sci Rep.

[47] Cuberovic I, Gill A, Resnik LJ, Tyler DJ, Graczyk EL (2019). Learning of artificial sensation through long-term home use of a sensory-enabled prosthesis. Front Neurosci.

[48] Desveaux L (2016). Physical activity in adults with diabetes following prosthetic rehabilitation. Can J Diabetes.

[49] Godfrey B, Berdan J, Kirk MN, Chou TR (2018). The accuracy and validity of modus Trex activity monitor in determining functional level in veterans with Transtibial amputations. J Prosthetics Orthot.

[50] Halsne EG, Waddingham MG, Hafner BJ (2013). Long-term activity in and among persons with transfemoral amputation. J Rehabil Res Dev.

[51] Kent JA, Stergiou N, Wurdeman SR (2015). Step activity and stride-to-stride fluctuations are negatively correlated in individuals with transtibial amputation. Clin Biomech.

[52] Lin S-J, Winston KD, Mitchell J, Girlinghouse J, Crochet K (2014). Physical activity, functional capacity, and step variability during walking in people with lower-limb amputation. Gait Posture.

[53] Mandel A (2016). Balance confidence and activity of community-dwelling patients with transtibial amputation. J Rehabil Res Dev.

[54] Orendurff MS, Kobayashi T, Villarosa CQ, Coleman KL, Boone DA (2016). Comparison of a computerized algorithm and prosthetists’ judgment in rating functional levels based on daily step activity in transtibial amputees. J Rehabil Assist Technol Eng.

[55] Orendurff MS (2016). Functional level assessment of individuals with transtibial limb loss: evaluation in the clinical setting versus objective community ambulatory activity. J Rehabil Assist Technol Eng.

[56] Parker K, Kirby RL, Adderson J, Thompson K (2010). Ambulation of people with lower-limb amputations: relationship between capacity and performance measures. Arch Phys Med Rehabil.

[57] Pepin M-E, Devour A, Coolsaet R, Galen S (2019). Correlation between functional ability and physical activity in individuals with Transtibial amputations. Cardiopulm Phys Ther J.

[58] Resnik L, Acluche F, Borgia M (2018). The DEKA hand: a multifunction prosthetic terminal device—patterns of grip usage at home. Prosthetics Orthot Int.

[59] Samuelsen BT (2017). The impact of the immediate postoperative prosthesis on patient mobility and quality of life after Transtibial amputation. Am J Phys Med Rehabil.

[60] Sanders JE (2018). Residual limb fluid volume change and volume accommodation: relationships to activity and self-report outcomes in people with trans-tibial amputation. Prosthetics Orthot Int.

[61] Sions JM, Arch ES, Horne JR (2018). Self-reported functional mobility, balance confidence, and prosthetic use are associated with daily step counts among individuals with a unilateral transtibial amputation. J Phys Act Health.

[62] Stepien JM, Cavenett S, Taylor L, Crotty M (2007). Activity levels among lower-limb amputees: self-report versus step activity monitor. Arch Phys Med Rehabil.

[63] Agrawal VR (2010). A comparison of gait kinetics between prosthetic feet during functional activities – symmetry in external work (SEW) approach. *ProQuest Dissertations and Theses*. (*University of Miami)*.

[64] Andrysek J (2017). Long-term clinical evaluation of the automatic stance-phase lock-controlled prosthetic knee joint in young adults with unilateral above-knee amputation. Disabil Rehabil Assist Technol.

[65] Berge JS, Czerniecki JM, Klute GK (2005). Efficacy of shock-absorbing versus rigid pylons for impact reduction in transtibial amputees based on laboratory, field, and outcome metrics. J Rehabil Res Dev.

[66] Buis AWP (2014). Measuring the daily stepping activity of people with transtibial amputation using the ActivPAL™ activity monitor. J Prosthetics Orthot.

[67] Christiansen CL (2018). Behavior-change intervention targeting physical function, walking, and disability after Dysvascular amputation: a randomized controlled pilot trial. Arch Phys Med Rehabil.

[68] Coleman KL, Boone DA, Laing LS, Mathews DE, Smith DG (2004). Quantification of prosthetic outcomes: elastomeric gel liner with locking pin suspension versus polyethylene foam liner with neoprene sleeve suspension. J Rehabil Res Dev.

[69] Darter BJ (2007). The effects of an integrated motor learning based treadmill mobility and aerobic exercise training program in persons with a transfemoral amputation. *ProQuest Dissertations and Theses*. (*University of Iowa*).

[70] Gailey RS (2012). Application of self-report and performance-based outcome measures to determine functional differences between four categories of prosthetic feet. J Rehabil Res Dev.

[71] Graczyk EL, Resnik L, Schiefer MA, Schmitt MS, Tyler DJ (2018). Home use of a neural-connected sensory prosthesis provides the functional and psychosocial experience of having a hand again. Sci Rep.

[72] Hafner BJ, Askew RL (2015). Physical performance and self-report outcomes associated with use of passive, adaptive, and active prosthetic knees in persons with unilateral, transfemoral amputation: randomized crossover trial. J Rehabil Res Dev.

[73] Hafner BJ, Willingham LL, Buell NC, Allyn KJ, Smith DG (2007). Evaluation of function, performance, and preference as Transfemoral amputees transition from mechanical to microprocessor control of the prosthetic knee. Arch Phys Med Rehabil.

[74] Highsmith MJ (2016). Effects of the Genium knee system on functional level, stair ambulation, perceptive and economic outcomes in Transfemoral amputees. Technol Innov.

[75] Highsmith MJ, Kahle JT, Quillen WS, Mengelkoch LJ (2012). Spatiotemporal parameters and step activity of a specialized stepping pattern used by a transtibial amputee during a denali mountaineering expedition. J Prosthetics Orthot.

[76] Hsu M-J, Nielsen DH, Lin-Chan S-J, Shurr D (2006). The effects of prosthetic foot design on physiologic measurements, self-selected walking velocity, and physical activity in people with transtibial amputation. Arch Phys Med Rehabil.

[77] Klute GK, Berge JS, Orendurff MS, Williams RM, Czerniecki JM (2006). Prosthetic intervention effects on activity of lower-extremity amputees. Arch Phys Med Rehabil.

[78] Klute GK (2011). Vacuum-assisted socket suspension compared with pin suspension for lower extremity amputees: effect on fit, activity, and limb volume. Arch Phys Med Rehabil.

[79] Klute GK, Bates KJ, Berge JS, Biggs W, King C (2016). Prosthesis management of residual-limb perspiration with subatmospheric vacuum pressure. J Rehabil Res Dev.

[80] Larson ER (2015). Massage therapy effects in a long-term prosthetic user with fibular hemimelia. J Bodyw Mov Ther.

[81] Littman AJ, Haselkorn JK, Arterburn DE, Boyko EJ (2019). Pilot randomized trial of a telephone-delivered physical activity and weight management intervention for individuals with lower extremity amputation. Disabil Health J.

[82] Morgan SJ (2018). Laboratory- and community-based health outcomes in people with transtibial amputation using crossover and energy-storing prosthetic feet: a randomized crossover trial. PLoS One.

[83] Raschke SU (2015). Biomechanical characteristics, patient preference and activity level with different prosthetic feet: a randomized double blind trial with laboratory and community testing. J Biomech.

[84] Sanders JE (2017). Effects of socket size on metrics of socket fit in trans-tibial prosthesis users. Med Eng Phys.

[85] Segal AD, Kracht R, Klute GK (2014). Does a torsion adapter improve functional mobility, pain, and fatigue in patients with transtibial amputation?. Clin Orthop Relat Res.

[86] Sherman K, Roberts A, Murray K, Deans S, Jarvis H (2019). Daily step count of British military males with bilateral lower limb amputations: a comparison of in-patient rehabilitation with the consecutive leave period between admissions. Prosthetics Orthot Int.

[87] Theeven PJ (2012). Influence of advanced prosthetic knee joints on perceived performance and everyday life activity level of low-functional persons with a transfemoral amputation or knee disarticulation. J Rehabil Med.

[88] Wurdeman SR, Schmid KK, Myers SA, Jacobsen AL, Stergiou N (2017). Step activity and 6-minute walk test outcomes when wearing low-activity or high-activity prosthetic feet. Am J Phys Med Rehabil.

[89] Arch ES (2016). Real-world walking performance of individuals with lower-limb amputation classified as Medicare functional classification level 2 and 3. J Prosthetics Orthot.

[90] Bussmann JB, Grootscholten EA, Stam HJ (2004). Daily physical activity and heart rate response in people with a unilateral transtibial amputation for vascular disease. Arch Phys Med Rehabil.

[91] Chadwell A, Kenney L, Thies S, Galpin A, Head J (2016). The reality of myoelectric prostheses: understanding what makes these devices difficult for some users to control. Front Neurorobot.

[92] Chadwell A (2019). Upper limb activity of twenty myoelectric prosthesis users and twenty healthy anatomically intact adults. Sci data.

[93] Chu CKG, Wong MS (2016). Comparison of prosthetic outcomes between adolescent transtibial and transfemoral amputees after Sichuan earthquake using step activity monitor and prosthesis evaluation questionnaire. Prosthetics Orthot Int.

[94] Hordacre B, Barr C, Crotty M (2015). Community activity and participation are reduced in transtibial amputee fallers: a wearable technology study. BMJ Innov.

[95] Hordacre B, Barr C, Crotty M (2014). Use of an activity monitor and GPS device to assess community activity and participation in transtibial amputees. Sensors (Basel).

[96] Paxton RJ, Murray AM, Stevens-Lapsley JE, Sherk KA, Christiansen CL (2016). Physical activity, ambulation, and comorbidities in people with diabetes and lower-limb amputation. J Rehabil Res Dev.

[97] Teknomo K (2014). Visualizing gait patterns of able bodied individuals and Transtibial amputees with the use of Accelerometry in smart phones. Rev Colomb Estadística.

[98] Smith JD, Guerra G, Burkholder BG (2019). The validity and accuracy of wrist-worn activity monitors in lower-limb prosthesis users. Disabil Rehabil.

[99] Hsu M-J (2002). Efficacy of energy storing-releasing prosthetic feet in individuals with transtibial amputation during ambulation: physiological, functional, and temporal/distance assessments. *ProQuest Dissertations and Theses*. (*University of Iowa*).

[100] Pearson EJM (2009). Comfort and its measurement - a literature review. Disabil Rehabil Assist Technol.

[101] Makin TR (2013). Deprivation-related and use-dependent plasticity go hand in hand. Elife.

[102] Lang CE, Waddell KJ, Klaesner JW, Bland MD (2017). A method for quantifying upper limb performance in daily life using accelerometers. J Vis Exp.

[103] Sobuh M, Kenney L, Tresadern P, Twiste M, Thies S (2010). Monitoring of upper limb prosthesis activity in trans-radial amputees. *Amputation, Prosthesis Use, and Phantom Limb Pain*. *Springer*.

[104] Phillips S, Curham K, Carey S (2012). Development of quality of use monitor for upper extremity prostheses. *RESNA Annual Conference*.

[105] Denaro BA, Schoenberg JS, Self BP, Bagley A (2001). Prosthetic arm monitoring system using a programmable interface controller. Biomed Sci Instrum.

[106] Bussmann HB, Reuvekamp PJ, Veltink PH, Martens WL, Stam HJ (1998). Validity and reliability of measurements obtained with an ‘activity monitor’ in people with and without a transtibial amputation. Phys Ther.

[107] Salih SA, Peel NM, Burgess K (2016). Monitoring activity of inpatient lower limb prosthetic users in rehabilitation using accelerometry: Validation study. J Rehabil Assist Technol Eng.

[108] Ramstrand N, Nilsson K-AÅ (2007). Validation of a patient activity monitor to quantify ambulatory activity in an amputee population. Prosthetics Orthot Int.

[109] Singleton J, Darcy S (2013). ‘Cultural life’, disability, inclusion and citizenship: moving beyond leisure in isolation. Ann Leis Res.

[110] Jaeger P (2005). Understanding disability: inclusion, access, diversity, and civil rights. Greenwood publishing group.

[111] Suyi Yang E, Aslani N, McGarry A (2019). Influences and trends of various shape-capture methods on outcomes in trans-tibial prosthetics: a systematic review. Prosthetics Orthot Int.

[112] Williams RJ, Takashima A, Ogata T, Holloway C (2019). A pilot study towards long-term thermal comfort research for lower-limb prosthesis wearers. Prosthetics Orthot Int.

[113] De Vries SI, Garre FG, Engbers LH, Hildebrandt VH, Van Buuren S (2011). Evaluation of neural networks to identify types of activity using accelerometers. Med Sci Sports Exerc.

[114] Redfield MT, Cagle JC, Hafner BJ, Sanders JE (2013). Classifying prosthetic use via accelerometry in persons with transtibial amputations. J Rehabil Res Dev.

[115] Knaier R, Höchsmann C, Infanger D, Hinrichs T, Schmidt-Trucksäss A (2019). Validation of automatic wear-time detection algorithms in a free-living setting of wrist-worn and hip-worn ActiGraph GT3X+. BMC Public Health.

[116] Bullock IM, Feix T, Dollar AM (2015). The Yale human grasping dataset: grasp, object, and task data in household and machine shop environments. Int J Robot Res.

[117] Healy A, Farmer S, Pandyan A, Chockalingam N (2018). A systematic review of randomised controlled trials assessing effectiveness of prosthetic and orthotic interventions. PLoS One.

[118] Chadwell A (2018). The reality of myoelectric prostheses: how do EMG skill, unpredictability of prosthesis response, and delays impact on user functionality and everyday prosthesis use? (*PhD thesis, University of Salford*).

[119] Condie E, Scott H, Treweek S (2006). Lower limb prosthetic outcome Measures : a review of the literature 1995 to 2005. J Prosthetics Orthot.

[120] Ostler C (2018). The me-amputee study: exploring meaningful outcomes of recovery following lower limb amputation and prosthetic rehabilitation: the patient’s perspective. In *ISPO UK Annual Scientific Meeting*.

[121] Kett M, Cole E, Turner J (2020). Disability, mobility and transport in low- and middle-income countries: a thematic review. Sustainability..

[122] Mckechnie PS, John A (2014). Anxiety and depression following traumatic limb amputation: a systematic review. Injury..

[123] Marzano L (2015). The application of mHealth to mental health: opportunities and challenges. Lancet Psychiatry.

[124] Aranda-Jan C, Boutard A. *Understanding the mobile disability gap*. London: GSM Association; 2019.

[125] Sexton S. *Rehabilitation of people with physical disabilities in developing countries*. Brussels: International Society for Prosthetics and Orthotics; 2016.

[126] Groce N, et al. Poverty and disability – a critical review of the literature in low and middle-income countries. *Leonard Cheshire Research Centre Working Paper Series: No. 16*. London: University College London; 2011.

[127] Paul G, Irvine J (2014). Privacy implications of wearable health devices. Proceedings of the 7th international conference on security of information and networks.

[128] Goyal R, Dragoni N, Spognardi A (2016). Mind the tracker you wear - a security analysis of wearable health trackers. Proceedings of the ACM symposium on applied computing.

[129] Day MC, Wadey R, Strike S (2019). Living with limb loss: everyday experiences of “good” and “bad” days in people with lower limb amputation. Disabil Rehabil.

[130] McMullan C, Wilkes S (2019). A study in Progress: sensory preference in prosthetics.

[131] Earnshaw VA (2018). Stigma-based bullying interventions: a systematic review. Dev Rev.

[132] Deans S, Burns D, McGarry A, Murray K, Mutrie N (2012). Motivations and barriers to prosthesis users participation in physical activity, exercise and sport: a review of the literature. Prosthetics Orthot Int.

[133] Wernke MM (2017). Progress toward optimizing prosthetic socket fit and suspension using elevated vacuum to promote residual limb health. Adv Wound Care.

[134] Laitala K, Klepp I, Henry B (2017). Global laundering practices – alternatives to machine washing. Househ Pers Care Today.

[135] Mahajan A, Pottie G, Kaiser W (2020). Transformation in healthcare by wearable devices for diagnostics and guidance of treatment. ACM Trans Comput Healthc.

